# Haplotype-resolved and chromosome-level reference genome assembly of *Diospyros deyangensis* provides insights into the evolution and juvenile growth of persimmon

**DOI:** 10.1093/hr/uhaf001

**Published:** 2025-01-08

**Authors:** Changfei Guan, Yunxiao Liu, Zhongxing Li, Yangxin Zhang, Zhiguang Liu, Qinggang Zhu, Pingxian Zhang, Xiaoxia Shen, Jing Fang, Jiayan Li, Qingling Zhang, Qingmei Guan, Zhengrong Luo, Yong Yang, Tao Zhao

**Affiliations:** Department of Horticulture, Hainan Institute of Northwest A&F University, Sanya 572024, China; State Key Laboratory for Crop Stress Resistance and High-Efficiency Production, College of Horticulture, Northwest A&F University, Yangling, Shaanxi, 712100, China; Department of Horticulture, Hainan Institute of Northwest A&F University, Sanya 572024, China; State Key Laboratory for Crop Stress Resistance and High-Efficiency Production, College of Horticulture, Northwest A&F University, Yangling, Shaanxi, 712100, China; Department of Horticulture, Hainan Institute of Northwest A&F University, Sanya 572024, China; College of Grassland Agriculture, Northwest A&F University, Yangling, Shaanxi, 712100, China; Department of Horticulture, Hainan Institute of Northwest A&F University, Sanya 572024, China; State Key Laboratory for Crop Stress Resistance and High-Efficiency Production, College of Horticulture, Northwest A&F University, Yangling, Shaanxi, 712100, China; Department of Horticulture, Hainan Institute of Northwest A&F University, Sanya 572024, China; State Key Laboratory for Crop Stress Resistance and High-Efficiency Production, College of Horticulture, Northwest A&F University, Yangling, Shaanxi, 712100, China; Department of Horticulture, Hainan Institute of Northwest A&F University, Sanya 572024, China; State Key Laboratory for Crop Stress Resistance and High-Efficiency Production, College of Horticulture, Northwest A&F University, Yangling, Shaanxi, 712100, China; Agricultural Genomics Institute at Shenzhen, Chinese Academy of Agricultural Sciences, Shenzhen, Guangdong, 518000, China; Department of Horticulture, Hainan Institute of Northwest A&F University, Sanya 572024, China; College of Grassland Agriculture, Northwest A&F University, Yangling, Shaanxi, 712100, China; Department of Horticulture, Hainan Institute of Northwest A&F University, Sanya 572024, China; State Key Laboratory for Crop Stress Resistance and High-Efficiency Production, College of Horticulture, Northwest A&F University, Yangling, Shaanxi, 712100, China; Department of Horticulture, Hainan Institute of Northwest A&F University, Sanya 572024, China; State Key Laboratory for Crop Stress Resistance and High-Efficiency Production, College of Horticulture, Northwest A&F University, Yangling, Shaanxi, 712100, China; National Key Laboratory for Germplasm Innovation & Utilization of Horticultural Crops, College of Horticulture and Forestry Sciences, Huazhong Agricultural University, Wuhan 430070, Hubei Province, China; Department of Horticulture, Hainan Institute of Northwest A&F University, Sanya 572024, China; State Key Laboratory for Crop Stress Resistance and High-Efficiency Production, College of Horticulture, Northwest A&F University, Yangling, Shaanxi, 712100, China; National Key Laboratory for Germplasm Innovation & Utilization of Horticultural Crops, College of Horticulture and Forestry Sciences, Huazhong Agricultural University, Wuhan 430070, Hubei Province, China; Department of Horticulture, Hainan Institute of Northwest A&F University, Sanya 572024, China; State Key Laboratory for Crop Stress Resistance and High-Efficiency Production, College of Horticulture, Northwest A&F University, Yangling, Shaanxi, 712100, China; Department of Horticulture, Hainan Institute of Northwest A&F University, Sanya 572024, China; State Key Laboratory for Crop Stress Resistance and High-Efficiency Production, College of Horticulture, Northwest A&F University, Yangling, Shaanxi, 712100, China

## Abstract

The *Diospyros* genus , which includes both wild and cultivated species such as *Diospyros lotus* and *Diospyros kaki*, represents a diverse genetic pool with significant agricultural value. In this study, we present a high-quality, haplotype-resolved, chromosome-level genome assembly for *Diospyros deyangensi*s (hereinafter referred to as ‘Deyangshi’), an autotetraploid wild species notable for its short juvenile phase, by integrating high-fidelity single-molecule, nanopore sequencing, and high-throughput chromosome conformation capture techniques. The assembled genome size is ~3.01 Gb, anchored onto 60 pseudochromosomes. Comparative genomic analysis revealed that the *D. deyangensis* genome underwent an additional whole-genome duplication (WGD) event following the eudicots shared ancient hexaploidy event. Resequencing and clustering on 63 samples representing 11 geographically diverse *Diospyros* accessions revealed significant genetic differentiation between *D. deyangensis* and *D. kaki*, as well as between *D. kaki* and other *Diospyros* species using population genomic analyses, suggesting that *D. kaki* followed an independent evolutionary pathway. Additionally, we identified *DdELF4* (EARLY FLOWERING 4) from the ‘Deyangshi’ backcross population using bulked segregant RNA sequencing (BSR-seq) with 50 early-flowering and 50 non-early-flowering individuals. Overexpression of *DdELF4* in *Arabidopsis* resulted in delayed flowering and downregulation of FT gene expression, indicating its role as a flowering repressor. This high-quality genome assembly of ‘Deyangshi’ provides an essential genomic resource for the *Diospyros* genus, particularly for breeding programs focused on developing early-flowering persimmon varieties.

## Introduction

The *Diospyros* genus, part of the Ebenaceae family, encompasses >500 species distributed worldwide [1, 2]. Among these, cultivated persimmon (*Diospyros kaki* Thunb.) stands out as a fruit crop of substantial nutritional value and economic importance. Persimmons are particularly notable for their high accumulation of proanthocyanidins, bioactive compounds with promising medicinal properties [3, 69]. Presently, persimmon cultivation is widespread in East Asia, South America, and the Mediterranean regions, underscoring its global agricultural relevance [4]. Cultivated persimmon is predominantly a hexaploid species (2n = 6x = 90) with high heterozygosity [5], posing challenges for investigating its genetic inheritance. Phylogenetic studies suggest that *D. kaki* originated in China [1, 6], but its progenitor(s) remain poorly understood due to its predominantly hexaploidy nature.

*Diospyros deyangensis*, commonly known as ‘Honghua Yemao’ persimmon or ‘Deyangshi’ in Chinese, is a perennial, dioecious, and deciduous woody plant native to Deyang City in Sichuan Province, China. This species exhibits distinct morphological characteristics that set it apart from other members of the *Diospyros* genus. Chromosomal analysis and flow cytometry studies confirm that *D. deyangensis* possesses 60 chromosomes, establishing it as a tetraploid species [7]. In addition, Sequence-Related Amplified Polymorphism (SRAP) molecular markers have shown that *D. deyangensis* has a distant genetic relationship with other persimmon species, suggesting that it could represent a unique lineage within the *Diospyros* genus [7]. Supporting this hypothesis, Guan *et al.* [8] utilized transcriptomic data from nine *Diospyros* species and found that *D. deyangensis* shared a closer genetic relationship with *D. kaki*. This positioning implies that *D. deyangensis* might serve as a wild relative of the cultivated persimmon (*D. kaki)*, with no evidence of complex hybridization events in its lineage. This relationship highlights the species' potential as a genetic reservoir for persimmon breeding programs, especially for traits beneficial to cultivated varieties.

One of the most remarkable characteristics of *D. deyangensis* is its drastically shortened juvenile phase, reaching the flowering stage in just 1–2 years, compared to the typical 5–8 years for most persimmon species. Notably, in contrast to mature specimens, it is the terminal buds of the seedlings that produce flowers, which are characterized by pale yellow petals [9]. As a perennial fruit tree, *D. deyangensis* exhibits an unusually rapid transition from vegetative to reproductive growth. This trait not only enriches our understanding of juvenile phases in fruit trees but also presents promising applications in breeding programs aimed at reducing the juvenile period in persimmons, as well as in functional gene validation efforts.

In this study, we present a high-quality, haplotype-resolved, chromosome-level genome assembly of *D. deyangensis*. Additionally, we performed whole-genome resequencing on a diverse set of 63 samples across 11 *Diospyros* species, covering both wild and cultivated varieties. By investigating genetic diversity and evolutionary relationships, we aim to better understand the unique lineage of *D. deyangensis* and its genetic divergence from *D. kaki*, the cultivated persimmon. To explore the genetic basis for this early-flowering trait, we utilized bulked segregant RNA sequencing (BSR-seq) on a backcross population of *D. deyangensis*, identifying candidate genes linked to juvenility. These findings lay the groundwork for genome-assisted breeding strategies aimed at developing short-juvenile persimmon cultivars, enhancing breeding efficiency within the *Diospyros* genus.

## Results

### Genome assembly and phasing

**Figure 1:**
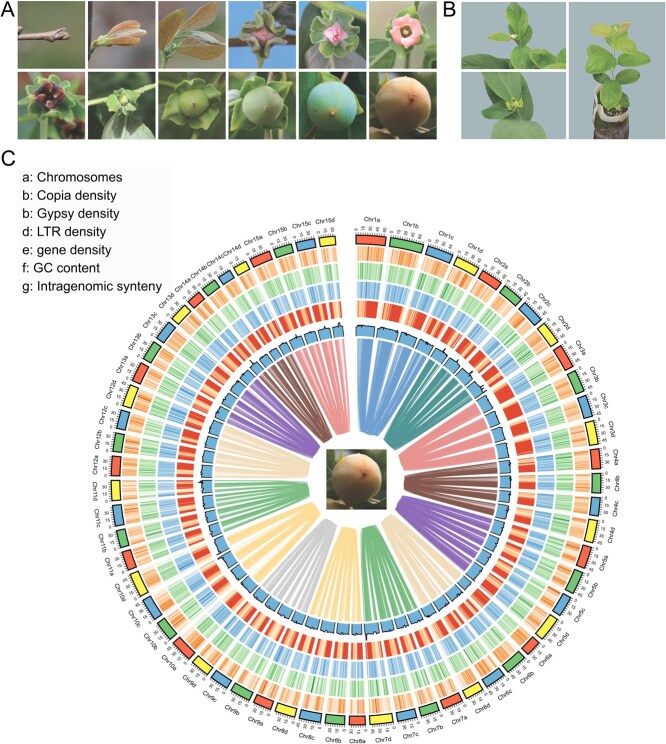
The morphology and homologous genome blocks for *D. deyangensis*. **(A)** Primary phenological growth stages of *D. deyangensis* including bud development stage, leaf development stage, flowering stage, and fruit development stage. **(B)** Flowering, setting fruit, and growing conditions in the first year of sowing. **(C)** Circos plot of major genomic characteristics. The outmost greenish circle comprises 60 pseudochromosomes. The features from outside to inside are (a) Chromosomes, (b) Copia density, (c) Gypsy density, (d) long terminal repeat retrotransposon density, (e) gene density, (f) GC content, and (g) the intrasubgenome syntenic regions. All distributions are drawn in a window size of 1 Mb. Syntenic blocks are represented by the inner lines. Each line represents a syntenic block.

We assembled the genome of *D. deyangensis* (‘Deyangshi’), an ancient tetraploid species (2n = 4x = 60), which has been proposed to play a role in the evolution of *D. kaki* (Fig. 1a, b)*.* Initial Illumina paired-end read sequencing generated 95.07 Gb of data, from which we estimated the genome size of *D. deyangensis* to be ~2.903 Gb with a heterozygosity rate of 3.2%, as predicted by GenomeScope2 using *k*-mer distribution analysis (Fig. S1).

To achieve a high-quality assembly, we employed multiple sequencing technologies: PacBio (Pacific Biosciences) HiFi (High-Fidelity), ONT (Oxford Nanopore Technology) long reads, and Hi-C (High-throughput chromosome conformation capture). We generated 81.05 Gb of HiFi long reads using the PacBio Revio platform (Fig. S2; Table S1). For further scaffolding, we incorporated 202 Gb of paired-end Illumina reads from a Hi-C library, enhancing the contiguity and structure of the assembly (Table S2). Additionally, ~155 Gb of nanopore sequencing reads (~53× coverage) were generated, providing an N50 of 28.42 kb for long-read data (Table S3). Using the Hifiasm assembler, this dataset produced an initial assembly of 3.01 Gb across 3089 contigs with an N50 of 13.13 Mb, closely matching the estimated genome size from *k*-mer analysis (Table 1).

**Table 1:** Summary for the ‘Deyangshi’ genome assembly and annotation

Categories	Size
Total length of genome assembly (Gb)	3.01
Assembly size (Mb)	657.04 (Hap A)
	668.14 (Hap B)
	657.58 (Hap C)
	677.06 (Hap D)
Number of chromosomes	60
Number of gaps	292
Anchor rate (%)	88.32%
N50 contig length (Mb)	13.13
N50 scaffold length (Mb)	43.72
GC content (%)	32.52
Repetitive sequences (%)	55.54
Number of annotated genes	29 398
	28 585
	29 264
	29 872
Number of gene models	234 374
Gene length (Mb)	683 958
Mean gene length (bp)	5839.61
Total CDS length (Mb)	149.972

Leveraging Hi-C interaction signals, we anchored 2.66 Gb (88.32%) of the contig sequences onto 60 pseudochromosomes, organized into 15 groups, each representing four homologous chromosomes (Fig. 1c). The final genome assembly measured ~3.01 Gb with an improved N50 value of 43.72 Mb (Table S4; S5), and the assembled genome displayed a GC content of 32.52% (Fig. 1c).

To assess the quality of the *D. deyangensis* genome assembly, we conducted a Benchmarking Universal Single-Copy Orthologs (BUSCO) analysis. The results indicate that each of the four monoploid genomes of *D. deyangensis* (each consisting of 15 chromosomes) contains 91.70%, 89.40%, 89.10%, and 84.10% of complete BUSCO genes, respectively. When combined, the assembly includes 97.5% of the complete BUSCO genes, with 2268 out of 2326 conserved genes identified from the eudicots_db10 database (Fig. S3; Table S6). For comparison, a prior study on autotetraploid cultivated alfalfa (also comprised of multiple monoploid genomes) reported slightly lower BUSCO completeness percentages (88.50%, 88.30%, 87.50%, 87.20%, and an overall completeness of 97.2%) [10]. Further evaluation of assembly quality through the Hi-C contact matrix confirmed clear and distinct chromosome groupings, reflecting well-organized chromosomal structure (Fig. 1c). Additionally, the Long Terminal Repeat Assembly Index (LAI) was measured at >14, indicating that the quality of the *D. deyangensis* assembly meets reference standards (Fig. S4; Table S6).

**Figure 2:**
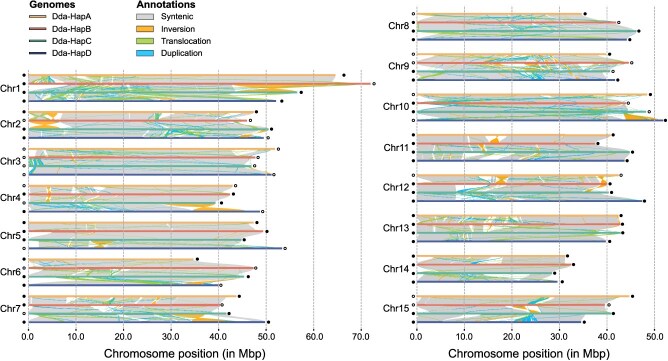
Comparative analysis of genome synteny and structural variations among the A, B, C, and D haplotypes of *D. deyangensis* genomes. Alignments were constructed using minimap2 and NUCMER. SyRI was used to analyze variations. Syntenic regions, inversions, translocations, and duplications are highlighted by different colors. Circles at both ends of the chromosome are used to represent the situation of telomeres. The solid means that the telomere is detected.

We also annotated telomeres within the assembled genome, identifying a total of 82 telomeric regions based on the conserved telomeric sequence (AAACCCT). Specifically, 33 chromosomes possess telomeres at both ends, while 16 chromosomes have telomeres at only one end (Fig. 2; Table S7). Overall, these results underscore the high quality, accuracy, and completeness of the *D. deyangensis* genome assembly, confirming its successful phasing into four distinct haplotypes.

### Genome annotation

For annotating the *D. deyangensis* genome, we employed three approaches: *ab initio* predictions, homology-based predictions, and RNA-seq analysis. This comprehensive annotation process yielded 117 119 protein-coding genes across the chromosome-level assembly. Each of the four haplotypes contributed as follows: haplotype A with 29 398 genes, haplotype B with 28 585 genes, haplotype C with 29 264 genes, and haplotype D with 29 872 genes (Table 1). We assessed the completeness of gene annotations using BUSCO method. Results revealed that the four monoploid genomes (each comprising 15 chromosomes) contained 88.10%, 85.70%, 86.10%, and 80.40% complete BUSCO genes, respectively. Collectively, 94.8% of the BUSCO genes (2204 out of 2326 conserved genes in the eudicots_db10 database) were present in the assembly (Fig. S5; Table S8). Furthermore, 97 071 collinear genes were identified, representing 82.88% of the predicted genes (Table S9).

### Evaluation of ploidy and haplotype structure

To further elucidate the polyploid origin of *D. deyangensis*, we conducted Smudgeplot analysis, which examined the depth and frequency of heterozygous *k*-mer pairs. The results showed that AAAB patterns predominated at 67%, while AABB accounted for only 17% (Fig. S6). This suggests a high degree of homology and low rediploidization, aligning with characteristics of other well-studied autotetraploid species such as *Medicago sativa* [10], *Selenicereus megalanthus* [11], *Acipenser sinensis* [1,2], and *Ziziphus mauritiana* [13].

Hi-C contact maps showed strong correlations among the four haplotypes of each chromosome, with each haplotype aligning closely to a single diploid chromosome, confirming their relatedness (Fig. S7–S9). Additionally, the distribution of synonymous substitutions per site (*Ks*) and gene length comparisons revealed no significant differences across the four haplotypes in the 15 chromosome sets (Fig. S10–S11). Across the monoploid genomes, 21 822 synteny clusters were identified, with 12 368 shared among all four genomes, 64 201 genes were identified within the shared synteny clusters. A total of 25 841 gene families were identified in the four monoploid genomes, 15 587 were common to all four monoploid genomes, and 70–214 were unique to individual monoploid genomes, underscoring high homologous gene retention (Fig. S12–S14).

Gene expression analysis in fruit, root, and leaf tissues across homologous chromosome groups showed no significant dominance among the haplotypes, indicating balanced gene expression profiles (Fig. S15–S17).

Phylogenetic analysis of the four haplotypes for each chromosome set supported the autotetraploid nature of *D. deyangensis*, as most homologous chromosome topologies favored an autotetraploid configuration (Fig. S18).

*K*-mer analysis revealed that the autotetraploid species exhibited a significantly lower number of unique *k*-mers compared to allotetraploid species, indicating low genomic divergence among monoploid genomes (Fig. S19).

Finally, using SubPhaser software, we were unable to phase the subgenomes based on subgenome-specific and repetitive *k*-mer differential signatures, which also indicates the genome may be autopolyploided [14].

Genomic comparison revealed a number of structural variations among the four haplotypes, such as duplications, inversions, and translocations, as well as deletions and insertions. In total, we have identified 8 531 487 single nucleotide polymorphisms (SNPs), 950 104 insertions/deletions (indels), 248 inversions, and 6842 translocations (Fig. 2).

### Comparative genomic analysis with *D. deyangensis*, *Diospyros oleifera*, and *D. lotus*

**Figure 3:**
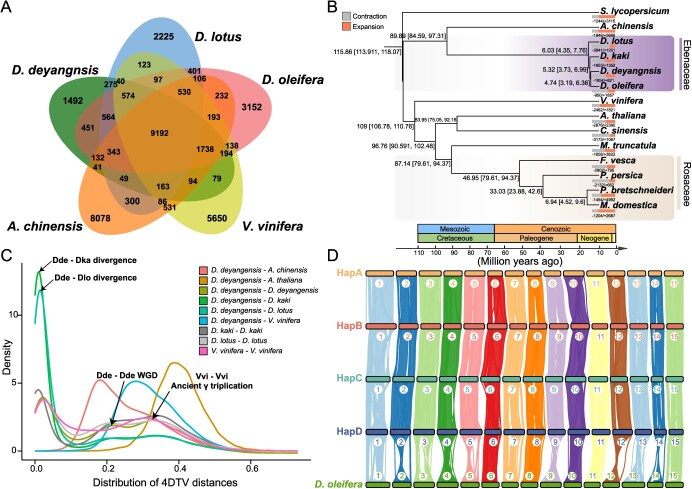
Comparative genomic analysis of *D. deyangensis*. (**A**) Venn diagram of orthologous gene families in *D. deyangensis*, *D. lotus*, *D. oleifera*, *V. vinifera*, and *A. chinensis*. The number of gene families are listed for each component. (**B**) A phylogenetic tree using single-copy gene families of *D. deyangensis* and other 13 species indicated. The proportion of expanded and contracted gene families is shown in orange and gray, respectively. Divergence times were estimated with the package PAML. The number next to the nodes represents the divergence time (million years ago). (**C**) The analysis of the 4DTv distance between orthologous syntenic genes among *D. deyangensis*, *D. lotus*, *D. oleifera*, *A. thaliana*, *V. vinifera*, and *A. chinensis*. The polyploidization events are referenced on the peaks. (**D**) Collinearity analysis between subgenomes of autotetraploid *D. deyangensis* and genome of diploid *D. oleifera***.**

Comparative genomic analysis of *D. deyangensis*, *D. oleifera*, and *D. lotus*, along with outgroups *Actinidia chinensis* and *Vitis vinifera*, identified 37 263 gene families, with 9192 shared among all species. *D. deyangensis*-specific genes were found in 1492 families, totaling 2089 (Fig. 3a; Table S10). Gene Ontology (GO) and Kyoto Encyclopedia of Genes and Genomes (KEGG) analyses highlighted unique genes in *D. deyangensis* involved in protein dimerization, transcription regulation, and postembryonic development (Fig. S20), and in alkaloid biosynthesis and terpene degradation pathways (Fig. S21; Table S11). Phylogenetic analysis using IQ-TREE placed *D. deyangensis* closely related to *D. oleifera*, with a divergence time in the Neogene epoch, indicating a recent common ancestor (Fig. 3b; Fig. S22). Whole-genome duplication (WGD) events were inferred from 4DTv and *Ks* distributions, suggesting a more recent event in *D. deyangensis* compared to *D. oleifera* and *D. lotus* (Fig. 3c; Fig. S23). A synteny dot plot further confirmed a recent WGD in *D. deyangensis* (Fig. S24). Additional analysis indicated an extra WGD event post the ancient γ-triploid event in *Diospyros* species (Fig. 3c; Fig. S24). Collinearity analysis between *D. deyangensis* and *D. oleifera* revealed 73 993 collinear genes, representing 50.11% of the total genes, underscoring evolutionary gene retention (Fig. 3d; Table S12).

### Genetic structure and diversity of 11 *Diospyros* species

**Figure 4:**
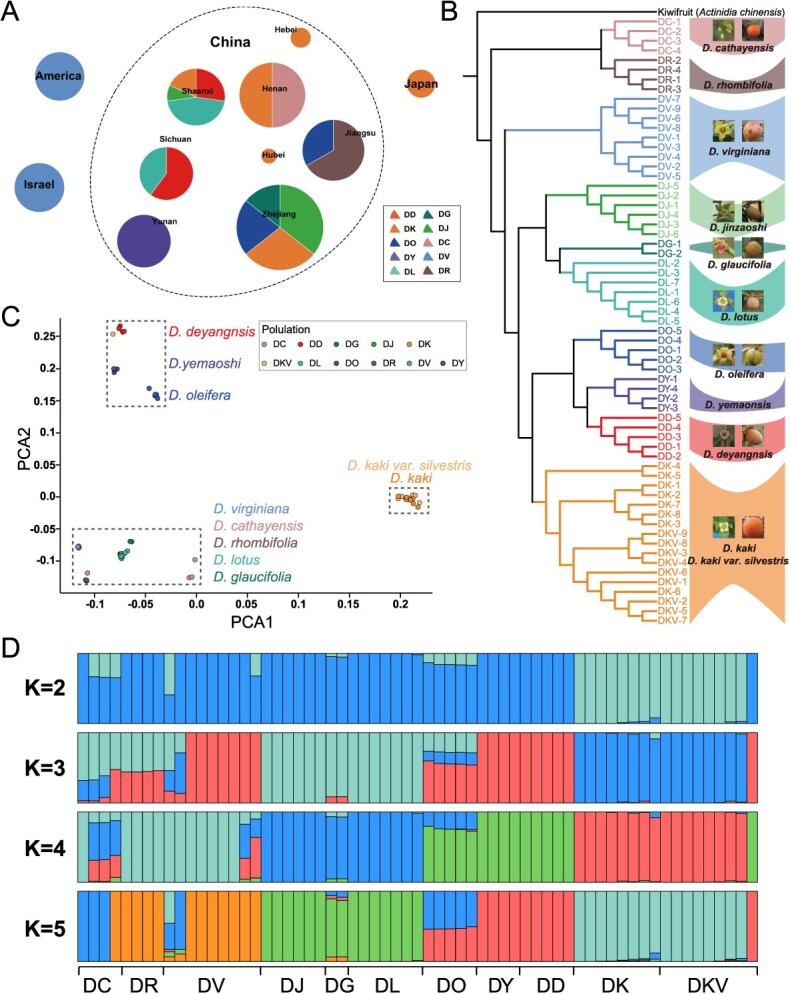
Genetic structure analysis of *Diospyros* samples. **(A)** Location of 63 *Diospyros* samples. **(B)** A mash-based phylogeny using Illumina raw reads of *Diospyros* and Actinidiaceae family species. Kiwifruit (*A. chinensis*) was used as the outgroup, with its Illumina resequencing data retrieved from Genome Sequence Archive (GSA) [15]. **(C)** PCA of all samples. **(D)** Admixture analysis of *Diospyros* samples. An ancestral population's proportion of the individual genome is represented by each colored segment. DD, *D. deyangensis*; DK, *D. kaki*; DKV, *D. kaki var. silvestris*; DO, *D. oleifera*; DV, *D. virginiana*; DC, *D. cathayensis*; DY, *D. yemaoensis*; DR, *D. rhombifolia*; DG, *D. glaucifolia*; DJ, *D. jinzaoshi*; DL, *D. lotus.*

To understand the genetic relationships within the *Diospyros* genus and clarify the origin of *D. kaki*, we sequenced 63 samples from 11 species across diverse geographical regions, including *D. deyangensis*, *D. kaki*, *D. kaki var. silvestris*, *D. oleifera*, *Diospyros virginiana*, *Diospyros cathayensis*, *Diospyros yemaoensis*, *Diospyros rhombifolia*, *Diospyros glaucifolia*, *Diospyros jinzaoshi*, and *D. lotus* (Fig. 4a; Table S13). We constructed a pangenome graph of *Diospyros* species based on the newly generated assemblies of *D. deyangensis* and published genomes representing major *Diospyros* genus. The total size of the constructed pangenome graph was 801 Mb. Notably, the core pangenome graph size was 406 Mb, representing 50.68% of the total pangenome size, suggesting that extensive divergent evolution has occurred among different *Diospyros* species.

Phylogenetic analysis revealed that *D. deyangensis*, *D. oleifera*, *D. yemaoensis*, *D. kaki var. silvestris,* and *D. kaki* formed a monophyletic clade, indicating shared ancestry among these species (Fig. 4b). Within this clade, it was challenging to distinguish *D. kaki* from its variant subspecies, *D. kaki var. silvestris*, based solely on genetic distance, suggesting a close genetic relationship. Additionally, the analysis showed that *D. cathayensis* and *D. rhombifolia* clustered separately, diverging earlier in the evolutionary timeline of the *Diospyros* genus.

Principal Component Analysis (PCA) supported these findings, with *D. deyangensis*, *D. oleifera*, and *D. yemaoensis* grouping closely together, while *D. kaki* and *D. kaki var. silvestris* formed a distinct yet related cluster, further indicating their genetic proximity (Fig. 4c).

Further exploration of population structure suggested a strong genetic homogeneity between *D. kaki* and *D. kaki var. silvestris*, distinguishing them from other species (Fig. 4d; Table S14). This implies that *D. kaki* did not originate from a hybridization event between *D. deyangensis* and *D. oleifera*, as previously hypothesized. Instead, the data support the theory that *D. kaki* likely emerged through the domestication of *D. kaki var. silvestris,* its ancestral subspecies.

### Identifying candidate loci for juvenility-growth by BSR-seq analysis

**Figure 5:**
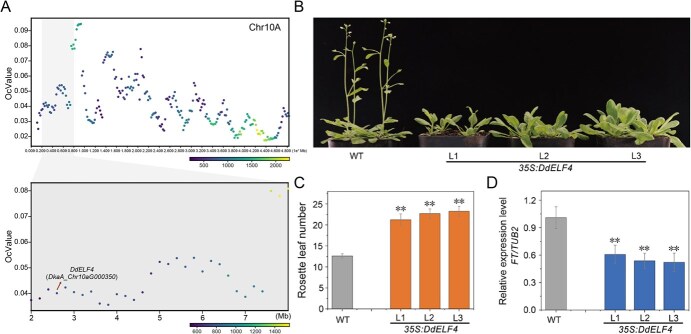
Candidate locus for early flowering in *D. deyangensis*. (A) QTL (quantitative trait loci) mapping results of OcBSA for the F_1_ populations of *D. deyangensis*. Scatter plots of OcBSA mapping signals for *D. deyangensis* populations. The color of the scatter plots indicates the number of SNPs/indels per Mb. (B) Phenotype of WT and 35S:*DdELF4* grown in long-day conditions. (C) Rosette leaf number of WT and 35S:*DdELF4* plants grown in long-day conditions. For each genotype, >15 plants were scored; bars indicate standard deviation; significant differences between the means of the genotypes were assessed using the two-tailed Student's *t*-test (**P < 0.01). (D) Relative expression level of *FT* in WT and 35S:*DdELF4*.

Previous studies have revealed that *D. kaki* seedlings typically take 5–8 years to flower, whereas *D. deyangensis* seedlings can flower in 1–2 years [7]. This disparity raises the mechanism underlying the transition from the juvenile phase to adult phase in ‘Deyangshi’. To gain insights into this process, we investigated candidate genes for the juvenility-growth trait in a backcross population of ‘Deyangshi’. Among the 399 BC1 seedlings, 349 plants did not flower, while 50 exhibited early flowering (Fig. S25). To identify the early-flowering loci in the *D. deyangensis*, we conducted BSR-seq using two bulk samples: one comprised of 50 early-flowering individuals (E-bulk) and the other 50 non-early-flowering individuals (N-bulk) from the BC1 segregants. We identified a candidate genomic region with early-flowering traits in ‘Deyangshi’ on Chr10A (Fig. 5a). By performing gene annotations and comparing them with the overlapped genes in model plants *Arabidopsis*, as well as considering expression level analysis, we pinpointed a flowering-related gene, *DdELF4*. In particular, *DdELF4* exhibited low expression levels in early-flowering seedlings (Table S15), suggesting its potential involvement in the transition from juvenile to adult phase in *D. deyangensis*.

Due to the limited availability of transgenic systems for *Diospyros* research, we chose to ectopically express *DdELF4* (35S:*DdELF4*) in *Arabidopsis* (Col-0 background). We then assessed the total number of leaves in 35S:*DdELF4* lines and compared them to the wild-type (WT) plants. The results displayed that 35S:*DdELF4* plants had a significantly higher total number of leaves compared to the WT plants (Fig. 5b and Fig. 5c). To further investigate the impact of *DdELF4* on flowering regulation, we examined the expression levels of a flowering-related gene, *FLOWER LOCUS T* (*FT*). In 35S:*DdELF4* lines, *FT* expression was downregulated compared to WT plants (Fig. 5d). These results indicate that *DdELF4* functioned as a repressor in the regulation of flowering in ‘Deyangshi’.

## Discussion

In this study, we focused on assembling the genome of the autotetraploid species *D. deyangensis*, which exhibits short-juvenility characteristics. *D. deyangensis* genome deciphering provides the basis for studying how the genus *Diospyros* species originated and evolved. In recent studies, the genomes of two diploid *Diospyros* species, *D. oleifera* (2n = 2x = 30) and *D. lotus* (2n = 2x = 30), two hexaploidy *Diospyros* species *D. kaki* cv. Taishu (2n = 6x = 90) and *D. kaki* cv. Xiaoguotianshi (2n = 6x = 90) have been sequenced and assembled to elucidate genome evolution within the *Diospyros* genus [16–20]. According to previous analyses of the chloroplast genomes, *D. oleifera* and *D. deyangensis* belong to the same group [21]. Based on our phylogenetic tree and synteny analysis, *D. deyangensis* and *D. oleifera* are closely related with a divergence time estimated at 6–40 Mya. While *D. oleifera* and *D. lotus* have been proposed as possible ancestral species of cultivated persimmon [16, 20], our analysis of the phylogeny and genetic structure of the *Diospyros* genus based on 63 samples indicates that *D. deyangensis*, *D. oleifera*, and *D. lotus* did not undergo genetic admixture events. This suggests that they may not directly serve as the ancestors of the cultivated persimmons.

Clear morphological differences exist between *D. cathayensis*, *D. rhombifolia*, and *D. kaki*. Phylogenetic tree and PCA indicated that *D. cathayensis* and *D. rhombifolia* form a lineage connected to the outgroup (kiwifruit), suggesting that they are relatively ancient species within the *Diospyros* genus. Genetic relationships analysis revealed that *D. deyangensis*, *D. oleifera*, and *D. yemaoensis* are most closely related to *D. kaki*, while other *Diospyros* species show a more distant relationship with *D. kaki*. Admixture events were observed within the *D. kaki* accessions, likely resulting from artificial hybridization and breeding practices. However, few gene flows occurred between *D. kaki* and other wild relatives, possibly due to the characteristic of parthenocarpy in persimmon.

Regulation of flowering time involves the integration of environmental stimuli, such as day length and temperature, with an endogenous development program. Flowering time is a complex trait influenced by internal factors, such as age and nutrition, as well as external factors, including temperature, photoperiod, and light quality. In persimmon seedlings, the time it takes to reach the flowering stage is typically prolonged, lasting between 5–8 years, which possess challenges for breeding advancements. However, the discovery of a new *Diospyros* species ‘Deyangshi’ with a short-juvenility growth has presented a significant breakthrough [7]. Despite this advancement, the precise mechanism that controls flowering time in persimmon remains unclear. Through the use of BSR-seq, we identified a potential gene, *DdELF4*, which played a crucial role in the regulation of the transition from the juvenile phase to the adult phase in persimmon. The discovery of *DdELF4* holds great promise in unraveling the regulatory mechanisms that govern underlying floral transition in this particular species, and it also possesses considerable potential in facilitating breeding efforts aimed at cultivating persimmon varieties with a shorter juvenile phase. Additionally, we identified a candidate gene (*DkaA_Chr10aG002050*) located on Chr10A in the 0.809 × 10^7^-Mb genomic region that exhibited a high OcValue and is associated with seed germination and seedling growth. Functional annotation and homology analysis with *Arabidopsis thaliana* revealed that *DkaA_Chr10aG002050* is homologous to the GAS2 gene, which is involved in gibberellin biosynthesis. Previous studies have shown that loss of GAS2 reduces the inhibitory effects of abscisic acid (ABA) on seed germination and seedling growth [22]. Based on these findings, we hypothesized that *DkaA_Chr10aG002050* may be involved in regulating the early-flowering trait in *D. deyangensis*. However, future functional experiments are required to validate this hypothesis.

In summary, this study has assembled a haplotype-resolved and chromosome-level reference genome of the autotetraploid species ‘Deyangshi’, thereby shedding light on the evolutionary processes that that have shaped the genome of *Diospyros*. Additionally, we have identified the candidate genes that are closely associated with short-juvenile growth. These findings provide valuable genetic resources for genome-based breeding strategies, facilitating genetic improvements and breeding of persimmons with a short-juvenile trait.

## Materials and methods

### Plant materials

All plant samples were planted and preserved in the National Field Genebank for Persimmon (NFGP), China. Most of the *Diospyros* samples originated from China, with a smaller proportion sourced from Japan, the Americas, and Israel. The early-flowering ‘Deyangshi’-MF06 (Deyangshi-01 × ‘Deyangshi’-M01) was crossed as a female parent to ‘Deyangshi’-01 in the NFGP. A total of 399 seedlings from the BC1 population, comprising 50 early-flowering plants and 349 nonflowering plants, were utilized in a study investigating the regulation of flowering time. Young leaves were harvested for genomic DNA (gDNA) extraction by using CTAB method [23].

### Library construction and sequencing

The pair-end (PE) sequencing library was constructed as described in Chen *et al.* [68]. PE libraries with ~500-bp insert sizes were sequenced with the Illumina HiSeq 4000 sequencer (Illumina, USA) by BioMarker Company (Beijing, China). In total, 95.07 Gb raw Illumina sequencing data were produced, which represent 32× genome coverage. The steps of nanopore library construction follow the steps in Jiao *et al.* [24]. Portions of the DNA were used to construct circular consensus sequencing (CCS) libraries, which were subsequently sequenced on a PacBio Sequel platform. We generated 81.05 Gb of highly accurate (>99%) HiFi reads. For Hi-C sequencing, the Hi-C fragment libraries with insert sizes ranging from 300 to 700 bp were generated as described in Rao *et al.* [25] and were subsequently sequenced using the Illumina platform.

### Genome size estimation

To generate an updated estimate of genome size, we calculated the 27 *k*-mers with KMC (v3.2.3) [26] software using the HiFi reads and estimated the genome characteristics using GenomeScope 2.0 (https://github.com/tbenavi1/genomescope2.0) (parameters: -k 27 -p 4) and Smudgeplot (https://github.com/KamilSJaron/smudgeplot) [27].

### Genome assembly

For the assembly of *D. deyangensis*, HiFi reads, Hi-C short reads, and ONT reads were used as a combined input for the genome assembler hifiasm (v0.19.8-r603) (https://github.com/chhylp123/hifiasm) with the default parameters [28, 29]. Hi-C data were incorporated into the assembly using hifiasm as described in the hifiasm documentation (https://hifiasm.readthedocs.io/en/latest/index.html). Gfatools (v0.4-r214-dirty) (https://github.com/lh3/gfatools) was used to convert sequence graphs in the GFA to fasta format. The contigs of the two primary haplotypes produced by hifiasm were merged and aligned to the reference genome assembly *D. oleifera* using Minimap2 (v2.24-r1122) [30] with the parameter of ‘-x asm20’ to determine their potential chromosomal group according to the longest alignment block length and mapping quality (MAPQ) of the paf file. We further aligned Hi-C reads to the hifiasm assembly (merge of two primary haplotypes) using bwa mem (v0.7.17-r1188), filtered out polymerase chain reaction (PCR) duplicates using samblaster (v0.1.26), converted sam file to bam file using samtools view (parameters: -S -h -bq 1 -F 3340). Overlapped contigs (allelic contigs) were generated by analyzing the paf file, and the corresponding bam alignments files were filtered. The particular bam alignments file of contigs in each chromosome group was extracted, respectively. For each chromosome group, YaHS (v1.1a-r3) (https://github.com/c-zhou/yahs) [31] (parameters: --no-contig-ec -q 10) was used to scaffold the contigs assembly into chromosomes. The visualization of contigs and scaffolds was achieved using ALLHiC_plot (parameters: -m 500 k -s 1000 k) (v0.9.8) [32]. Following manual inspection and correction of misassemblies identified through abnormal Hi-C interaction matrices, four haplotypes for each chromosome group were manually distinguished according to the Hi-C interaction signals, and 60 pseudochromosomes were constructed. The chromosome orientations were confirmed through genome-wide alignment with previously published genomes. The telomeric sequences in the *D. deyangensis* genome assembly were identified using quarTeT toolkit (v1.1.3) (parameters: -c plant) [33]. The normalized and unified seven-base telomere repeat sequence AAACCCT within 50 kb of each terminal chromosome sequence was identified as a telomere.

### Genome quality evaluation

The assembled genome was analyzed using BUSCO (v5.7.1) [34] with the eudicots_db10 database to evaluate genome completeness. Long terminal repeat (LTR) structures were identified, and complete LTR elements were used to calculate the LAI value with the default parameters [35].

### Genome annotation

Within the genome sequence of *D. deyangensis*, repeat elements were identified *de novo* with the Extensive *de novo* TE Annotator (EDTA) pipeline (v2.0.0) [36]. The resulting annotations were used to build a high-quality nonredundant repeat sequence library. The nonredundant repeat sequence library was integrated with the Repbase database, and repetitive sequences were further masked using RepeatMasker (v4.1.2) [37]. To accurately identify LTR retrotransposons, LTR-retriever software [35] was used to integrate the outputs from LTRharvest [38] and LTR_FINDER [39] with default parameters.

We employed three approaches for protein-coding gene prediction in the Deyangshi genome: *ab initio* gene prediction, homology-based gene prediction using the MAKER2 pipeline [40], and RNA-seq-based gene prediction. PacBio Iso-Seq data was retrieved in the NCBI database (https://www.ncbi.nlm.nih.gov/sra/?term=SRR18500463). For homology-based prediction, we used GeMoMa (v1.3.1) [41] with genome sequences and gff files of *A. thaliana* (https://www.ncbi.nlm.nih.gov/search/all/?term=GCF_000001735.4), *A. chinensis* (https://www.ncbi.nlm.nih.gov/search/all/?term=GCF_019202715.1), and *D. lotus* (https://www.ncbi.nlm.nih.gov/search/all/?term=GCF_014633365) as input files. The protein sequences were mapped to the genome using tblastn (v2.9.0), and the Exonerate tool was used to acquire exact intron and exon positions. For the *ab initio* prediction, the integration of predicted gene models was performed using AUGUSTUS (v3.5.0) [42]. The consensus gene set was finally obtained using all the above data and predictions. Subsequently, we combined and integrated the annotations (all gene models) using EVidenceModeler (EVM) (v1.11) [43] to generate the final prediction results from the above three sources (*ab initio*, homology-based prediction, and RNA-seq-based prediction). To evaluate the completeness of gene annotations, BUSCO (v5.7.1) [34] was used for evaluating the annotated gene set based on the eudicots_db10 database.

### Identification of intrachromosomal genomic variations

The four *D. deyangensis* haplotypes genomes were aligned to each other using NUCMER embedded in MUMmer (v4.0.0beta2) [44] program with parameters of ‘-c 90 -l 40’ and minimap2 (v2.24-r1122) [30] with parameters of ‘-ax asm5 —eqx’. The comparison identified syntenic regions, structural rearrangements (duplications, translocations, and inversions), and sequence differences (deletions and insertions) using SyRI software (v1.6) [45]. Plotsr toolkit (v0.5.5) was used to generate visualizations of synteny and structural rearrangements among the four haplotype genomes [46].

### Comparative genomic analysis

The protein sequence sets of 13 plant species were collected from *Solanum lycopersicum*, *A. chinensis*, *D. lotus*, *D. kaki*, *D. oleifera*, *V. vinifera*, *A. thaliana*, *Citrus sinensis*, *Medicago truncatula*, *Fragaria vesca*, *Prunus persica*, *Pyrus bretschneideri*, and *Malus domestica* (Table S16). Orthofinder (v2.4) [47] was utilized for identifying protein sequences for gene family classification. Functional enrichment analysis of the identified target genes was conducted using KEGG Orthology Based Annotation System (KOBAS, http://kobas.cbi.pku.edu.cn/) [48]. The phylogenetic analysis and alignment of coding sequences of common single-copy genes were conducted using IQ-TREE software (v1.6.11) [49]. MCMCTree, implemented in the phylogenetic analysis by maximum likelihood (PAML) package (v4.9) [50], was utilized to estimate the divergence time between *D. deyangensis* and other 13 species. To refine these estimates, we consulted the TimeTree database (http://www.timetree.org/) to obtain corrected species divergence times [50]. For whole-genome microsynteny-based phylogenetic inference, we used synteny matrix representation with a likelihood (Syn-MRL) pipeline (https://github.com/zhaotao1987/Syn-MRL) to reconstruct phylogenetic trees [51]. We used MIKE software (v1.0) [12] to construct the phylogenetic tree for four haplotypes of *D. deyangensis* and other *Diospyros* species. The expansion and contraction of the gene families in *D. deyangensis* were analyzed by using CAFE software (v4.2.1) [52]. Gene family clustering results and estimated divergence times between species were utilized with the parameters: λ = 0.02 and *P-*value <.05. Pairwise *Ks* values for each syntenic gene pair were estimated using KaKs_Calculator2.0 [53] with model-averaged method. The synteny network method was employed for syntenic block identification, network construction, and community detection by using the SynNet-Pipeline (https://github.com/zhaotao1987/SynNet-Pipeline) [54]. An infomap implemented under the ‘igraph’ package was employed to detect communities within the synteny networks. Phylogenomic profiles were further produced by counting the number of syntenic genes in each genome for each synteny cluster. The visualization of genomic collinearity was performed using NGenomeSyn (v1.41) [55].

### Pangenome graph construction and PanGenie genotyping

Minigraph-Cactus is a comprehensive pipeline for variation graph construction, integrating Minigraph to create a structural variation graph and the Cactus base aligner to generate base-level pangenome graphs from a set of input assemblies, while embedding haplotype paths. We applied the Minigraph-Cactus pangenome pipeline [56] to construct the *Diospyros* pangenome graph based on the genome assembly (*D. kaki* cv. Taishu), six subgenome assemblies (*D. kaki* cv. Xiaoguotianshi), and four subgenome assemblies (*D. deyangensis*). The Minigraph-Cactus pangenome pipeline followed these steps: constructing the Minigraph GFA, mapping the genomes back to the Minigraph, creating the Cactus alignment and creating the VG indexes. After generating the variant panel from the *Diospyros* assemblies. We genotyped 63 *Diospyros* samples using the PanGenie pipeline (v3.0.1) [57]. Each VCF file was then merged using BCFtools (v1.9) [58].

### Population structure analysis

To minimize the effects of linkage disequilibrium (LD) on population structure, we pruned the SNP dataset using PLINK (v1.90) [59] with the parameter ‘--indep-pairwise 50 10 0.2,’ resulting in high-quality SNPs with low LD for further analysis. Additionally, we applied the following filtering criteria to extract high-quality variants: [16] loci with genotypes exhibiting read depth values <0.02 were removed; and [60] only sites with a minor allele frequency (MAF) ≥0.05 were retained. PCA was then performed using the pruned SNP data. Population structure was investigated using ADMIXTURE program (v1.3.0) [60], where 2–10 genetic group (*K*) values were predetermined and the population admixture result was visualized using the R package pophelper (v2.3.1) [61]. The genomic distances among 63 *Diospyros* samples were calculated using Mashtree (v1.3.0) with the following parameters: -mindepth 0 -numcpus 6 *FORWARD.fastq.gz > mashtree.dnd [62]. The output tree (.dnd) from Mashtree was visualized and annotated with the online tool iTOL v5 (https://itol.embl.de/) [63].

### Identification of genomic loci underlying early flowering by BSR-seq

The early-flowering seedlings (flowering within the first year, E-bulk) and nonflowering seedlings (N-bulk) from the BC1 population were prepared (Fig. S25). The E-bulk and N-bulk were constructed by pooling equal amounts of RNA from 50 seedlings in each group. BSR-seq was performed on an Illumina HiSeq2500 platform using paired-end sequencing. Sequencing reads were trimmed and aligned to the ‘Deyangshi’ reference genome assembly using HISAT2 [64]. SNP calling was conducted with the HaplotypeCaller function in the GATK best practices pipeline. The final filtered SNPs from the early-flowering and nonflowering samples were used for subsequent analyses. BSR-seq and QTL mapping were performed using OcBSA [65].

### Overexpression of *DdELF4* in *Arabidopsis* and RT-qPCR assay

The coding sequences of *DdELF4* (*DkaA_Chr10aG000350*) were cloned and inserted into the expression vector pCambia2300-GFP (Table S17). The modified vector was transformed into Columbia *Col-0* (*A. thaliana*) using the floral dip method [66], and T3 homozygous lines were obtained as described in our previous study [9]. Total RNA was extracted from 10-day-old *Arabidopsis* seedlings grown under LD conditions using the RNAprep Pure Plant Plus Kit (TIANGEN, Beijing); the RNA was then used for reverse-transcription into cDNA. Real-time quantitative PCR (RT-qPCR) was performed using the ABI 7500 System with SYBR Green PCR master mix, as described previously [67]. The primer pairs for *DdELF4* amplification were listed in Table S17.

### Analysis of flowering time

To measure flowering time in *A. thaliana*, we counted the total leaves number (rosette plus cauline leaves) when the inflorescence reached ~3–5 cm in height [67]. *Arabidopsis* seedlings were grown in soil under long-day (16 h of light:8 h of dark) conditions with a photosynthetic photon flux of ~120 μmol m^−2^ s^−1^ at 22°C–24°C. More than 15 plants were analyzed for each line.

## Supplementary Material

Web_Material_uhaf001

## Data Availability

The raw sequence reads of the ‘Deyangshi’ genome and the whole-genome resequenced reads of the 63 *Diospyros* samples have been deposited in NCBI SRA, with the accession number PRJNA861725. The genome assembly, annotation data, and pangenome graph have been deposited at Zenodo (https://doi.org/10.5281/zenodo.14501941).

## References

[1] Luo ZR, Wang RZ. Persimmon in China: domestication and traditional utilizations of genetic resources. Adv Hortic Sci. 2008;22:239–43

[2] Tang DL, Zhang QL, Xu LQ. et al. Number of species and geographical distribution of *Diospyros* L. (Ebenaceae) in China. Hortic Plant J. 2019;2:59–69

[3] Guan CF, Duan XY, Zhang QL. et al. *DkPK* genes promote natural deastringency in C-PCNA persimmon by up-regulating *DkPDC* and *DkADH* expression. Front Plant Sci. 2017;8:149 28243247 10.3389/fpls.2017.00149 PMC5303730

[4] Matheus JRV, de Andrade CJ, Miyahira RF. et al. Persimmon (*Diospyros kaki* L.): chemical properties, bioactive compounds and potential use in the development of new products-a review. Food Rev Int. 2022;38:384–401

[5] Zhuang D, Kitajima A, Ishida M. et al. Chromosome number of *Diospyros kaki* cultivars. J Jpn Soc Hortic Sci. 1990;59:289–97

[6] Kanzaki S . The origin and cultivar development of Japanese persimmon (*Diospyros kaki* Thunb.). Nippon Shokuhin Kagaku Kogaku Kaishi. 2016;63:328–30

[7] Zhang YF, Yang Y, Guo J. et al. Taxonomic status of “Deyangshi” based on chromosome number and SRAP markers. Sci Hortic. 2016;207:57–64

[8] Guan CF, Liu SY, Wang MK. et al. Comparative transcriptomic analysis reveals genetic divergence and domestication genes in *Diospyros*. BMC Plant Biol. 2019;19:227 31146695 10.1186/s12870-019-1839-2 PMC6543618

[9] Fang J, Long H, Wang Z. et al. Genome-wide analysis of the early flowering 4 (ELF4) gene family in short-juvenile persimmon “Deyangshi” (*Diospyros deyangensis*) and its role of *DdELF4-8* during flowering control. Sci Hortic. 2023;310:111736

[10] Chen H, Zeng Y, Yang Y. et al. Allele-aware chromosome-level genome assembly and efficient transgene-free genome editing for the autotetraploid cultivated alfalfa. Nat Commun. 2020;11:2494 32427850 10.1038/s41467-020-16338-x PMC7237683

[11] Zaman QU, Hui L, Nazir MF. et al. Chromosome-level genome assembly of autotetraploid *Selenicereus megalanthus* and gaining genomic insights into the evolution of trait patterning in diploid and polyploid pitaya species. 2024; bioRxiv 2024.2006.2023.600268

[12] Wang F, Wang Y, Zeng X. et al. MIKE: an ultrafast, assembly-, and alignment-free approach for phylogenetic tree construction. Bioinformatics. 2024;40:4 10.1093/bioinformatics/btae154 PMC10990684 38547397

[13] Guo M, Bi G, Wang H. et al. Genomes of autotetraploid wild and cultivated *Ziziphus mauritiana* reveal polyploid evolution and crop domestication. Plant Physiol. 2024;196:2701–20 39325737 10.1093/plphys/kiae512

[14] Jia KH, Wang ZX, Wang L. et al. SubPhaser: a robust allopolyploid subgenome phasing method based on subgenome-specific *k*-mers. New Phytol. 2022;235:801–9 35460274 10.1111/nph.18173

[15] Lu XM, Yu XF, Li GQ. et al. Genome assembly of autotetraploid *Actinidia arguta* highlights adaptive evolution and enables dissection of important economic traits. Plant Commun. 2024;5:100856 38431772 10.1016/j.xplc.2024.100856 PMC11211551

[16] Akagi T, Shirasawa K, Nagasaki H. et al. The persimmon genome reveals clues to the evolution of a lineage-specific sex determination system in plants. PLoS Genet. 2020;16:e1008566 32069274 10.1371/journal.pgen.1008566 PMC7048303

[17] Horiuchi A, Masuda K, Shirasawa K. et al. Ongoing rapid evolution of a post-Y region revealed by chromosome-scale genome assembly of a hexaploid monoecious persimmon (*Diospyros kaki*). Mol Biol Evol. 2023;40:7 10.1093/molbev/msad151 PMC10350624 37414545

[18] Mao W, Yao G, Wang S. et al. Chromosome-level genomes of seeded and seedless date plum based on third-generation DNA sequencing and Hi-C analysis. Forestry Research. 2021;1:0 10.48130/FR-2021-0009 PMC11524226 39524504

[19] Suo Y, Sun P, Cheng H. et al. A high-quality chromosomal genome assembly of *Diospyros oleifera* Cheng. GigaScience. 2020;9:1 10.1093/gigascience/giz164 PMC6964648 31944244

[20] Zhu QG, Xu Y, Yang Y. et al. The persimmon (*Diospyros oleifera* Cheng) genome provides new insights into the inheritance of astringency and ancestral evolution. Hortic Res. 2019;6:138 31871686 10.1038/s41438-019-0227-2 PMC6917749

[21] Li WQ, Liu YL, Yang Y. et al. Interspecific chloroplast genome sequence diversity and genomic resources in *Diospyros*. BMC Plant Biol. 2018;18:210 30257644 10.1186/s12870-018-1421-3 PMC6158880

[22] Liu H, Guo S, Lu M. et al. Biosynthesis of DHGA(12) and its roles in Arabidopsis seedling establishment. Nat Commun. 2019;10:1768 30992454 10.1038/s41467-019-09467-5 PMC6467921

[23] Chang S, Puryear J, Cairney J. A simple and efficient method for isolating RNA from pine trees. Plant Mol Biol Report. 1993;11:113–6

[24] Jiao F, Luo RS, Dai XL. et al. Chromosome-level reference genome and population genomic analysis provide insights into the evolution and improvement of domesticated mulberry (*Morus alba*). Mol Plant. 2020;13:1001–12 32422187 10.1016/j.molp.2020.05.005

[25] Rao SS, Huntley MH, Durand NC. et al. A 3D map of the human genome at kilobase resolution reveals principles of chromatin looping. Cell. 2014;159:1665–80 25497547 10.1016/j.cell.2014.11.021 PMC5635824

[26] Kokot M, Dlugosz M, Deorowicz S. KMC 3: counting and manipulating *k*-mer statistics. Bioinformatics. 2017;33:2759–61 28472236 10.1093/bioinformatics/btx304

[27] Ranallo-Benavidez TR, Jaron KS, Schatz MC. GenomeScope 2.0 and Smudgeplot for reference-free profiling of polyploid genomes. Nat Commun. 2020;11:1432 32188846 10.1038/s41467-020-14998-3 PMC7080791

[28] Cheng H, Concepcion GT, Feng X. et al. Haplotype-resolved de novo assembly using phased assembly graphs with hifiasm. Nat Methods. 2021;18:170–5 33526886 10.1038/s41592-020-01056-5 PMC7961889

[29] Cheng H, Jarvis ED, Fedrigo O. et al. Haplotype-resolved assembly of diploid genomes without parental data. Nat Biotechnol. 2022;40:1332–5 35332338 10.1038/s41587-022-01261-x PMC9464699

[30] Li H . Minimap2: pairwise alignment for nucleotide sequences. Bioinformatics. 2018;34:3094–100 29750242 10.1093/bioinformatics/bty191 PMC6137996

[31] Zhou C, McCarthy SA, Durbin R. YaHS: yet another Hi-C scaffolding tool. Bioinformatics. 2023;39:1 10.1093/bioinformatics/btac808 PMC9848053 36525368

[32] Zhang X, Zhang S, Zhao Q. et al. Assembly of allele-aware, chromosomal-scale autopolyploid genomes based on Hi-C data. Nat Plants. 2019;5:833–45 31383970 10.1038/s41477-019-0487-8

[33] Lin Y, Ye C, Li X. et al. quarTeT: a telomere-to-telomere toolkit for gap-free genome assembly and centromeric repeat identification. Hortic Res. 2023;10:uhad127 37560017 10.1093/hr/uhad127 PMC10407605

[34] Manni M, Berkeley MR, Seppey M. et al. BUSCO update: novel and streamlined workflows along with broader and deeper phylogenetic coverage for scoring of eukaryotic, prokaryotic, and viral genomes. Mol Biol Evol. 2021;38:4647–54 34320186 10.1093/molbev/msab199 PMC8476166

[35] Ou S, Chen J, Jiang N. Assessing genome assembly quality using the LTR assembly index (LAI). Nucleic Acids Res. 2018;46:e126 30107434 10.1093/nar/gky730 PMC6265445

[36] Ou SJ, Su WJ, Liao Y. et al. Benchmarking transposable element annotation methods for creation of a streamlined, comprehensive pipeline. Genome Biol. 2019;20:275 31843001 10.1186/s13059-019-1905-y PMC6913007

[37] Tarailo-Graovac M, Chen N. Using RepeatMasker to identify repetitive elements in genomic sequences. Curr Protoc Bioinformatics. 2009;25:4.10.11–14.10.14 10.1002/0471250953.bi0410s25 19274634

[38] Ellinghaus D, Kurtz S, Willhoeft U. LTRharvest, an efficient and flexible software for de novo detection of LTR retrotransposons. BMC Bioinformatics. 2008;9:18 18194517 10.1186/1471-2105-9-18 PMC2253517

[39] Xu Z, Wang H. LTR_FINDER: an efficient tool for the prediction of full-length LTR retrotransposons. Nucleic Acids Res. 2007;35:W265–8 17485477 10.1093/nar/gkm286 PMC1933203

[40] Holt C, Yandell M. MAKER2: an annotation pipeline and genome-database management tool for second-generation genome projects. BMC Bioinformatics. 2011;12:491 22192575 10.1186/1471-2105-12-491 PMC3280279

[41] Keilwagen J, Hartung F, Grau J. GeMoMa: homology-based gene prediction utilizing intron position conservation and RNA-seq data. Methods Mol Biol. 2019;1962:161–77 31020559 10.1007/978-1-4939-9173-0_9

[42] Stanke M, Keller O, Gunduz I. et al. AUGUSTUS: ab initio prediction of alternative transcripts. Nucleic Acids Res. 2006;34:W435–9 16845043 10.1093/nar/gkl200 PMC1538822

[43] Haas BJ, Salzberg SL, Zhu W. et al. Automated eukaryotic gene structure annotation using evidence modeler and the program to assemble spliced alignments. Genome Biol. 2008;9:R7 18190707 10.1186/gb-2008-9-1-r7 PMC2395244

[44] Marçais G, Delcher AL, Phillippy AM. et al. MUMmer4: a fast and versatile genome alignment system. PLoS Comput Biol. 2018;14:e1005944 29373581 10.1371/journal.pcbi.1005944 PMC5802927

[45] Goel M, Sun H, Jiao WB. et al. SyRI: finding genomic rearrangements and local sequence differences from whole-genome assemblies. Genome Biol. 2019;20:277 31842948 10.1186/s13059-019-1911-0 PMC6913012

[46] Goel M, Schneeberger K. Plotsr: visualizing structural similarities and rearrangements between multiple genomes. Bioinformatics. 2022;38:2922–6 35561173 10.1093/bioinformatics/btac196 PMC9113368

[47] Emms DM, Kelly S. OrthoFinder: phylogenetic orthology inference for comparative genomics. Genome Biol. 2019;20:1–14 31727128 10.1186/s13059-019-1832-y PMC6857279

[48] Bu D, Luo H, Huo P. et al. KOBAS-i: intelligent prioritization and exploratory visualization of biological functions for gene enrichment analysis. Nucleic Acids Res. 2021;49:W317–w325 34086934 10.1093/nar/gkab447 PMC8265193

[49] Nguyen LT, Schmidt HA, Haeseler A. et al. IQ-TREE: a fast and effective stochastic algorithm for estimating maximum-likelihood phylogenies. Mol Biol Evol. 2015;32:268–74 25371430 10.1093/molbev/msu300 PMC4271533

[50] Yang Z . PAML 4: phylogenetic analysis by maximum likelihood. Mol Biol Evol. 2007;24:1586–91 17483113 10.1093/molbev/msm088

[51] Zhao T, Zwaenepoel A, Xue JY. et al. Whole-genome microsynteny-based phylogeny of angiosperms. Nat Commun. 2021;12:3498 34108452 10.1038/s41467-021-23665-0 PMC8190143

[52] Han MV, Thomas GWC, Lugo-Martinez J. et al. Estimating gene gain and loss rates in the presence of error in genome assembly and annotation using CAFE 3. Mol Biol Evol. 2013;30:1987–97 23709260 10.1093/molbev/mst100

[53] Wang D, Zhang Y, Zhang Z. et al. KaKs_Calculator 2.0: a toolkit incorporating gamma-series methods and sliding window strategies. Genomics Proteomics Bioinformatics. 2010;8:77–80 20451164 10.1016/S1672-0229(10)60008-3 PMC5054116

[54] Zhao T, Holmer R, de Bruijn S. et al. Phylogenomic synteny network analysis of MADS-box transcription factor genes reveals lineage-specific transpositions, ancient tandem duplications, and deep positional conservation. Plant Cell. 2017;29:1278–92 28584165 10.1105/tpc.17.00312 PMC5502458

[55] He W, Yang J, Jing Y. et al. NGenomeSyn: an easy-to-use and flexible tool for publication-ready visualization of syntenic relationships across multiple genomes. Bioinformatics. 2023;39:3 10.1093/bioinformatics/btad121 PMC10027429 36883694

[56] Hickey G, Monlong J, Ebler J. et al. Pangenome graph construction from genome alignments with Minigraph-Cactus. Nat Biotechnol. 2024;42:663–73 37165083 10.1038/s41587-023-01793-w PMC10638906

[57] Ebler J, Ebert P, Clarke WE. et al. Pangenome-based genome inference allows efficient and accurate genotyping across a wide spectrum of variant classes. Nat Genet. 2022;54:518–25 35410384 10.1038/s41588-022-01043-w PMC9005351

[58] Danecek P, Bonfield JK, Liddle J. et al. Twelve years of SAMtools and BCFtools. GigaScience. 2021;10:2 10.1093/gigascience/giab008 PMC7931819 33590861

[59] Purcell S, Neale B, Todd-Brown K. et al. PLINK: a tool set for whole-genome association and population-based linkage analyses. Am J Hum Genet. 2007;81:559–75 17701901 10.1086/519795 PMC1950838

[60] Alexander DH, Lange K. Enhancements to the ADMIXTURE algorithm for individual ancestry estimation. BMC Bioinformatics. 2011;12:246 21682921 10.1186/1471-2105-12-246 PMC3146885

[61] Francis RM . Pophelper: an R package and web app to analyse and visualize population structure. Mol Ecol Resour. 2017;17:27–32 26850166 10.1111/1755-0998.12509

[62] Katz LS, Griswold T, Morrison SS. et al. Mashtree: a rapid comparison of whole genome sequence files. J Open Source Softw. 2019;4:44 10.21105/joss.01762 PMC9380445 35978566

[63] Letunic I, Bork P. Interactive tree of life (iTOL) v5: an online tool for phylogenetic tree display and annotation. Nucleic Acids Res. 2021;49:W293–6 33885785 10.1093/nar/gkab301 PMC8265157

[64] Kim D, Langmead B, Salzberg SL. HISAT: a fast spliced aligner with low memory requirements. Nat Methods. 2015;12:357–60 25751142 10.1038/nmeth.3317 PMC4655817

[65] Zhang L, Duan Y, Zhang Z. et al. OcBSA: an NGS-based bulk segregant analysis tool for outcross populations. Mol Plant. 2024;17:648–57 38369755 10.1016/j.molp.2024.02.011

[66] Zhang XR, Henriques R, Lin SS. et al. *Agrobacterim*-mediated transformation of *Arabidopsis thaliana* using the floral dip method. Nat Protoc. 2006;1:641–6 17406292 10.1038/nprot.2006.97

[67] Zhang PX, Li XL, Wang YF. et al. PRMT6 physically associates with nuclear factor Y to regulate photoperiodic flowering in *Arabidopsis*. aBIOTECH. 2021;2:403–14 36304422 10.1007/s42994-021-00065-y PMC9590495

[68] Chen P, Li Z, Zhang D. et al. Insights into the effect of human civilization on *Malus* evolution and domestication. Plant Biotechnol J. 2021a;19:2206–20 34161653 10.1111/pbi.13648 PMC8541786

[69] Chen WX, Zheng QY, Li JW. et al. DkMYB14 is a bifunctional transcription factor that regulates the accumulation of proanthocyanidin in persimmon fruit. Plant J. 2021b;106:1708–27 33835602 10.1111/tpj.15266

