# Supplementary material for: Haplotype-resolved and chromosome-level reference genome assembly of Diospyros deyangensis provides insights into the evolution and juvenile growth of persimmon
Source: Hortic Res. 2025 Jan 8;12(4):uhaf001. doi: 10.1093/hr/uhaf001 (PMC11896977; doi:10.1093/hr/uhaf001)
Supplement: Web_Material_uhaf001 [file web_material_uhaf001.zip › Supplemental Figures-2024-12-26.pdf]

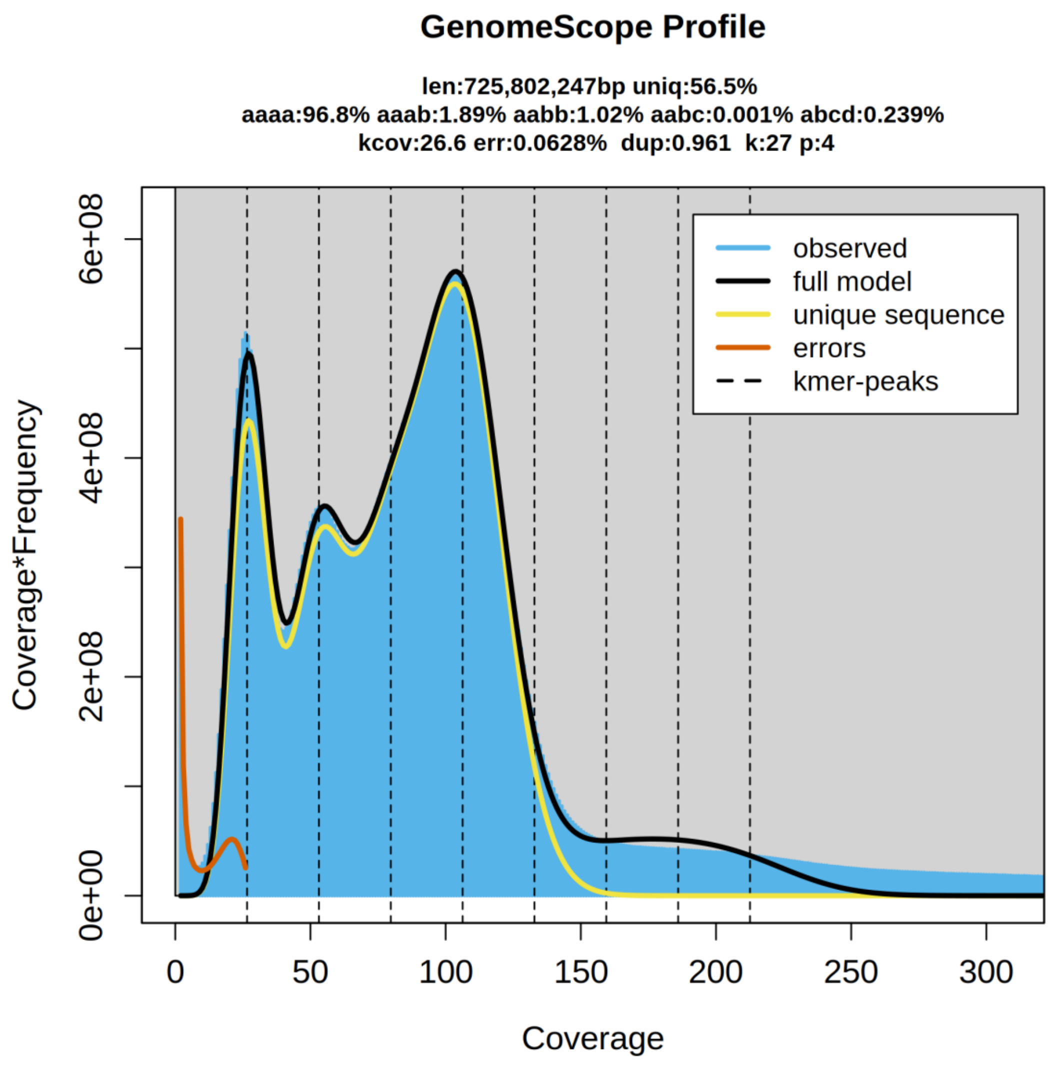

Supplemental Figure. S1. Genome survey for the genome of the haplotype *D. deyangensis* genome. The plot is generated using GenomeScope2.0 (k=27, ploidy=4). The X-axis is the depth, and the Y-axis is the proportion that exhibits the frequency at which depth is divided by the total frequency of all depths.

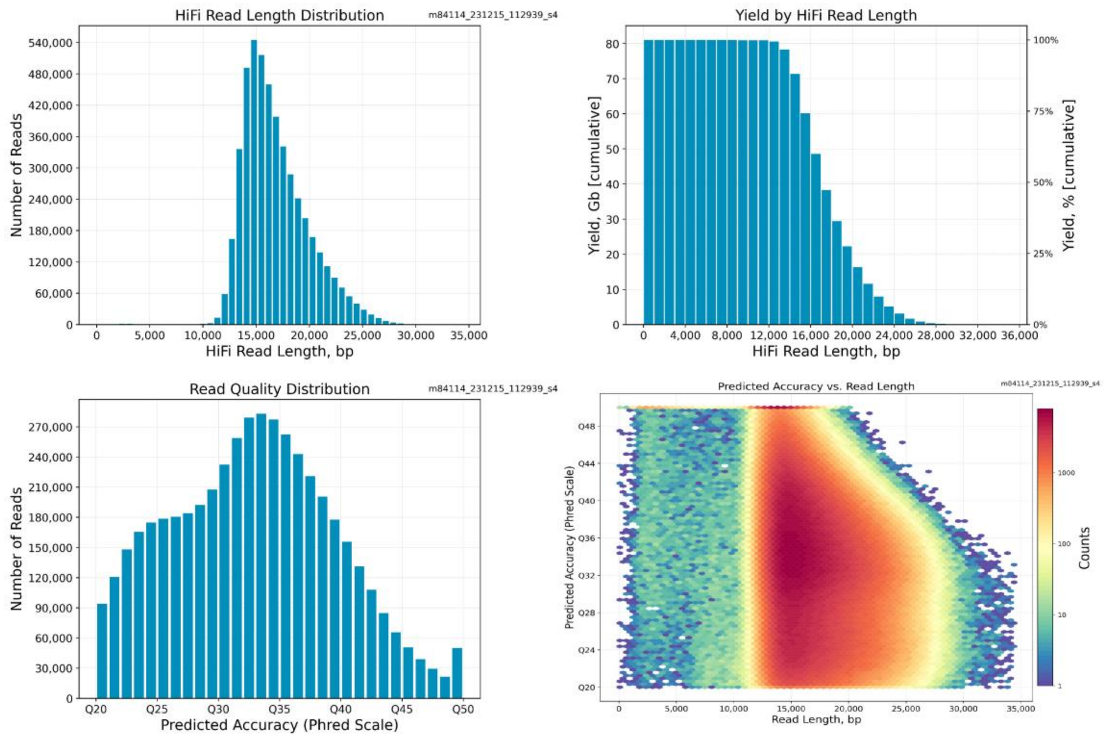

Supplemental Figure. S2. Summary of PacBio HiFi reads by PacBio Sequel II platform.

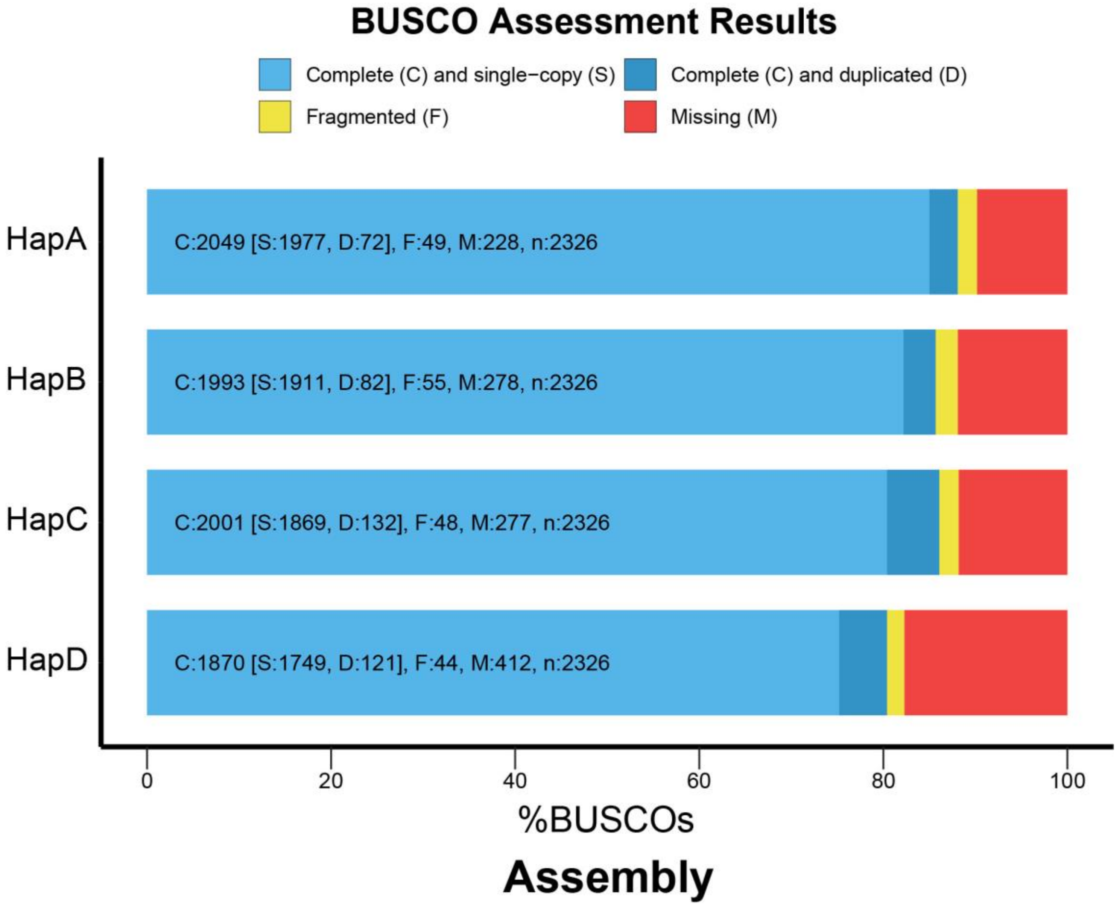

Supplemental Figure. S3. BUSCO assessment results of the assembly quality of haploid consensus genomes. Colors refer to the complete and single-copy orthologs (light blue), complete and duplicated orthologs (dark blue), fragmented orthologs (yellow), and missed orthologs (red). The listed numbers on the right of the chart show the percentage of complete orthologs (including both single-copy and duplicated).

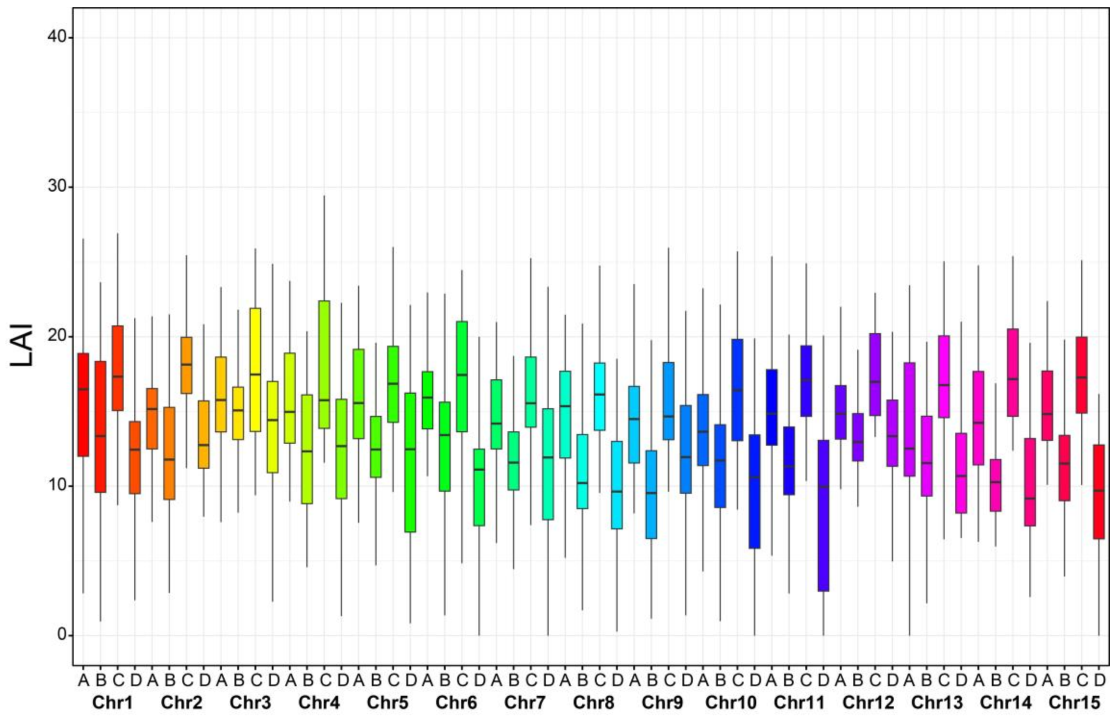

Supplemental Figure. S4. Evaluation of genome assemblies by LTR Assembly Index (LAI). The x-axes show the chromosomes of *D. deyangensis* genome.

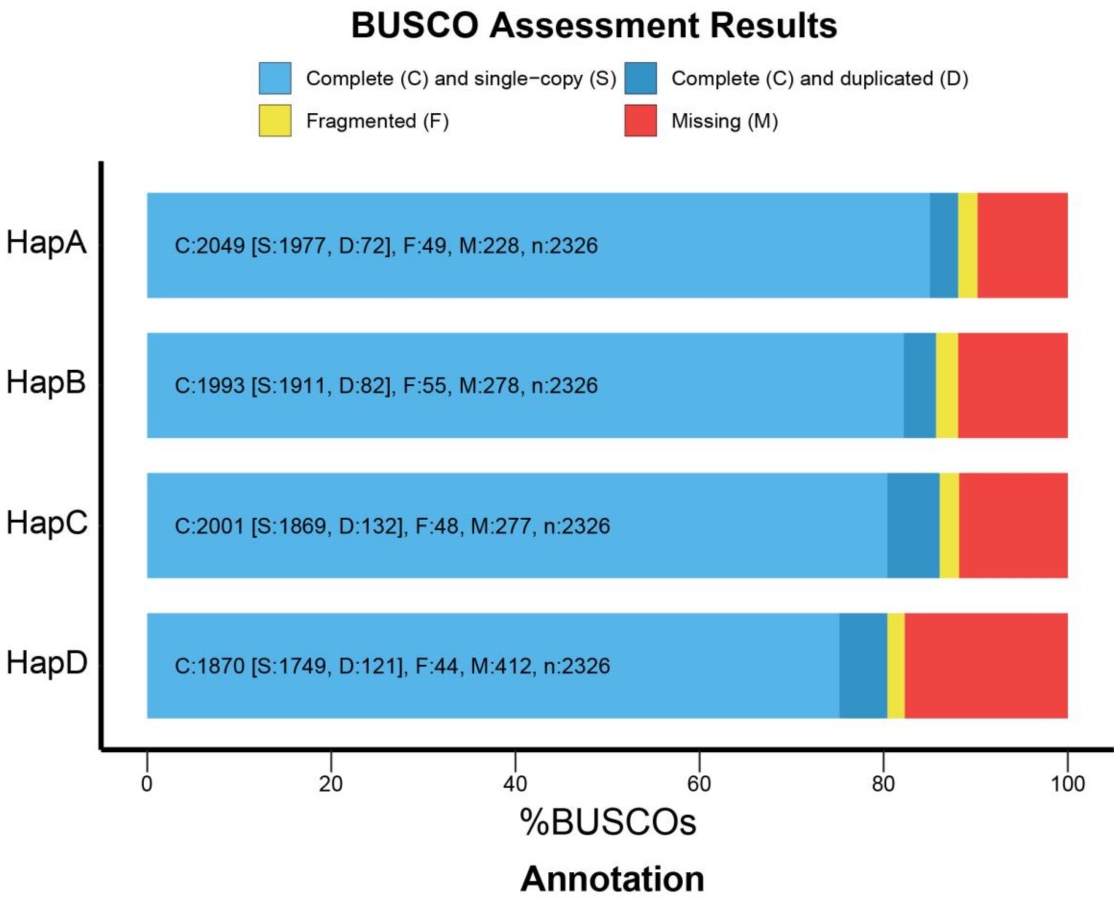

Supplemental Figure. S5. BUSCO assessment results of the annotation quality of haploid consensus genomes. Colors refer to the complete and single-copy orthologs (light blue), complete and duplicated orthologs (dark blue), fragmented orthologs (yellow), and missed orthologs (red). The listed numbers on the right of the chart show the percentage of complete orthologs (including both single-copy and duplicated).

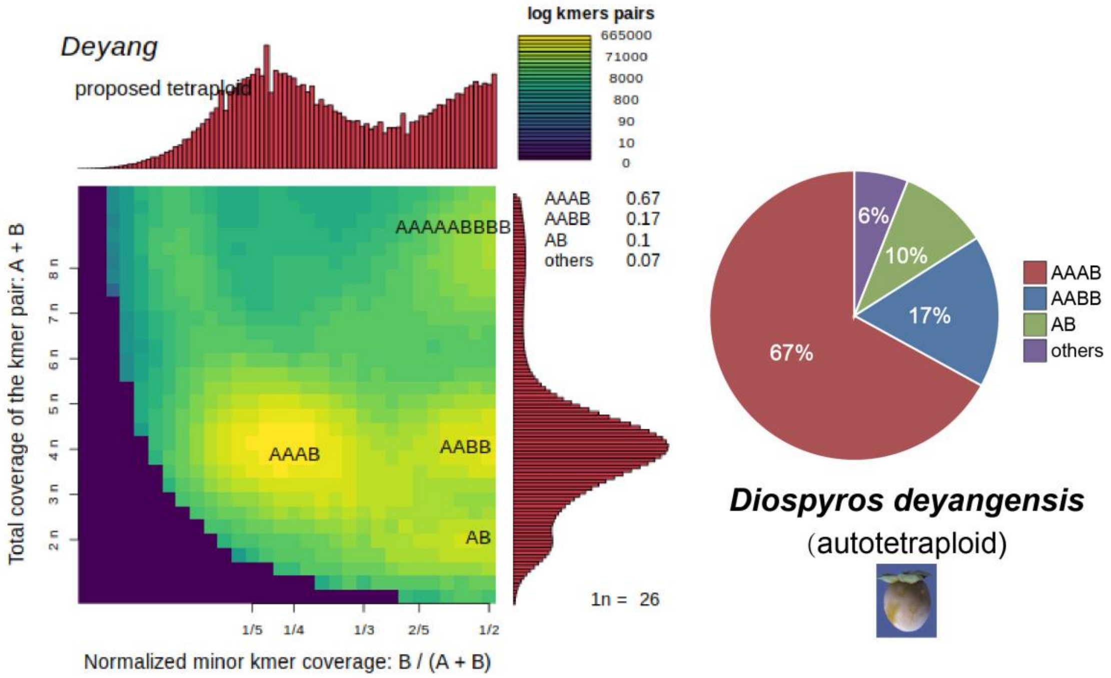

Supplemental Figure. S6 Ploidy compositions of *D. deyangensis*. In the smudgeplot of the polyploid species, the letters A and B represent a pair of heterozygous *k*-mers that differ by a single SNP. The intensity of each smudge reflects the number of heterozygous *k*-mers pairs contained within it. The estimated ploidy level is shown on the Y-Axis of the heatmap and various ploidies on the right side of the heatmap.

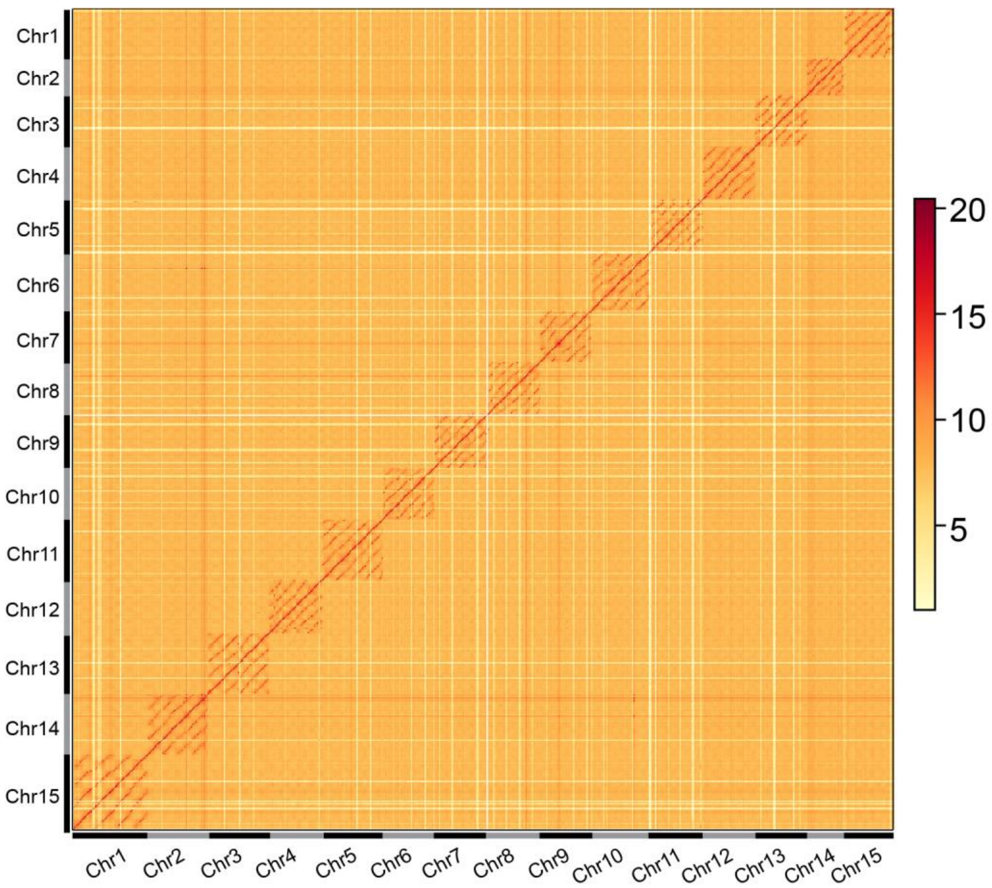

Supplemental Figure. S7. Overview of Hi-C interaction heatmap for assembled chromosomes of *D. deyangensis*. Each haplotype group contains four chromosomes.

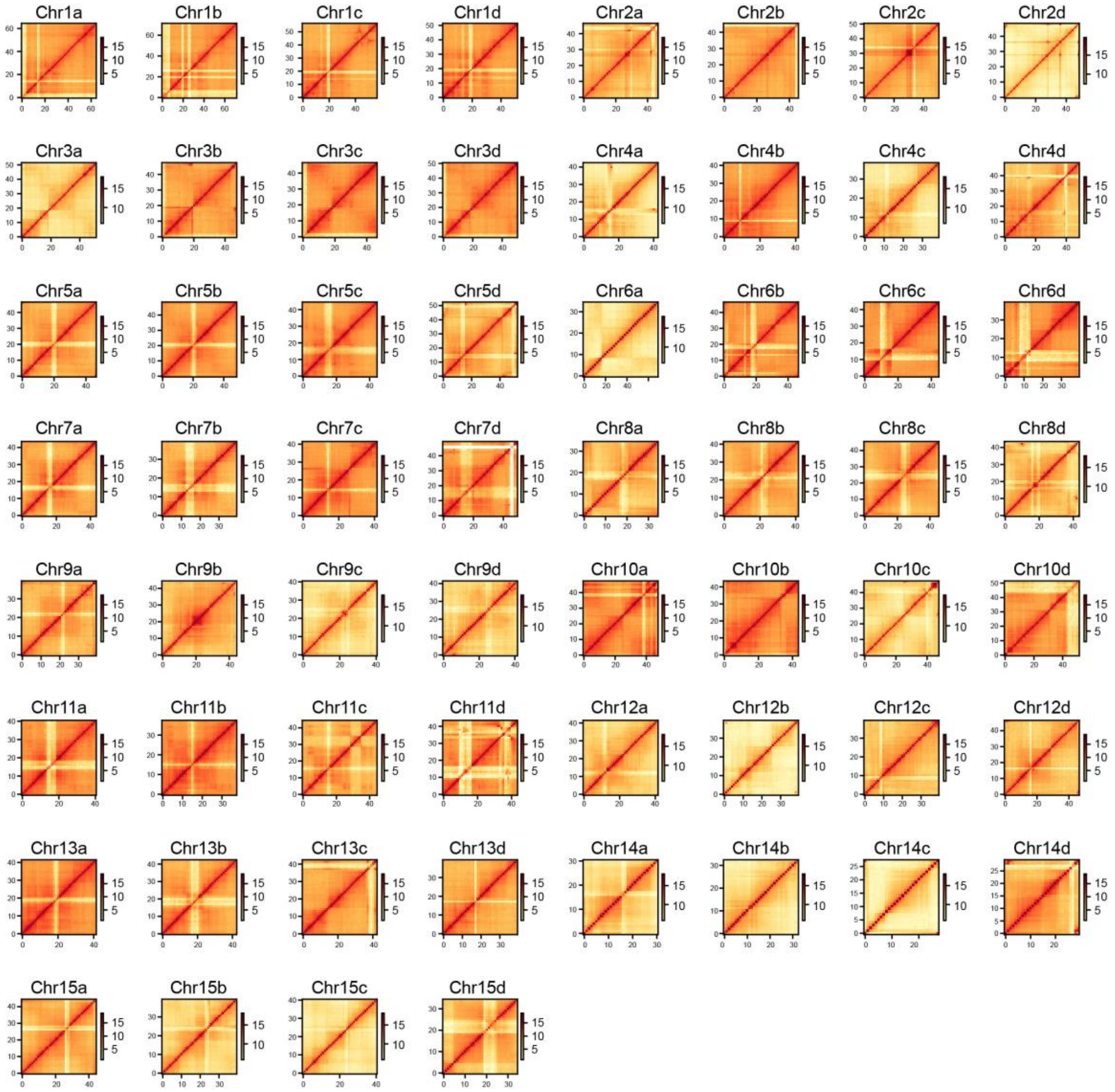

Supplemental Figure. S8. Hi-C intra-chromosomal contact map of 60 chromosomes for the genome assembly (4n=60) of *D. deyangensis*.

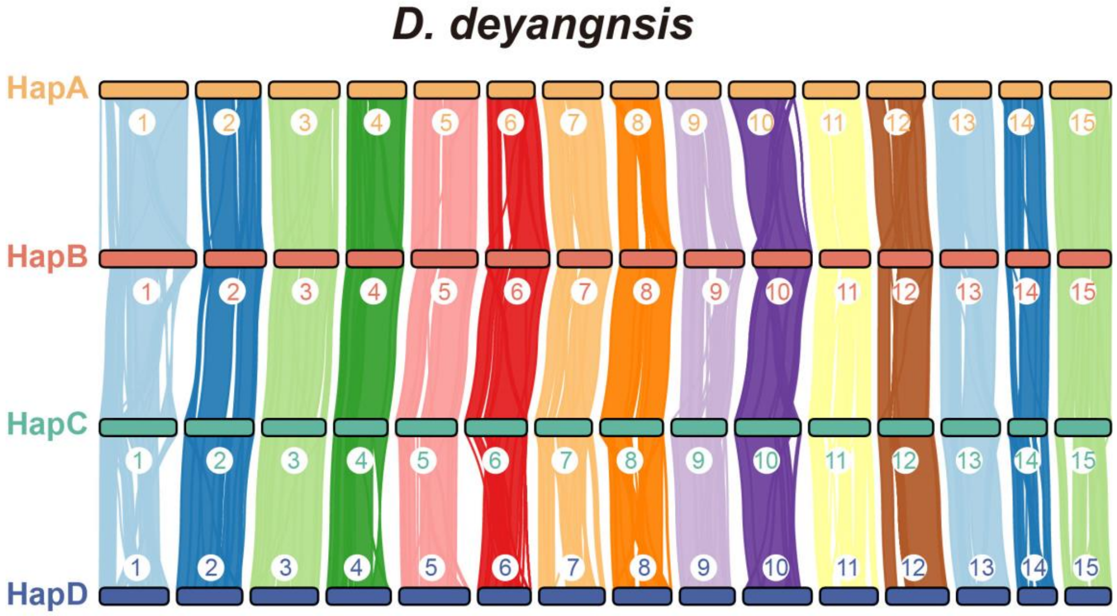

Supplemental Figure. S9. Genome synteny patterns of *D. deyangensis* (HapA, HapB, HapC, and HapD).

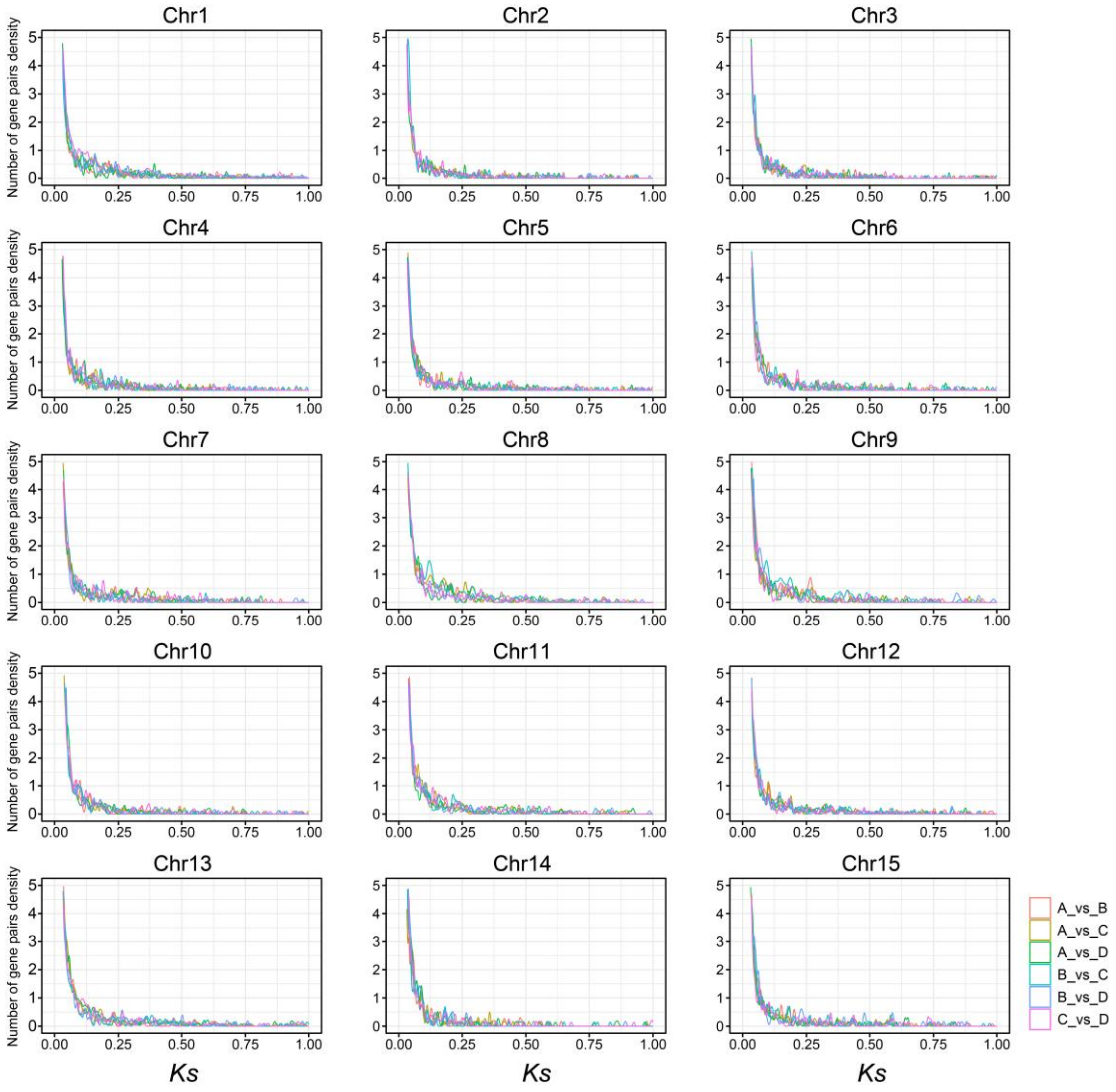

Supplemental Figure 10. The synonymous substitution rates (*Ks*) of syntenic gene pairs identified among four haplotypes (HapA, HapB, HapC, and HapD) in each chromosome groups of *D. deyangensis*.

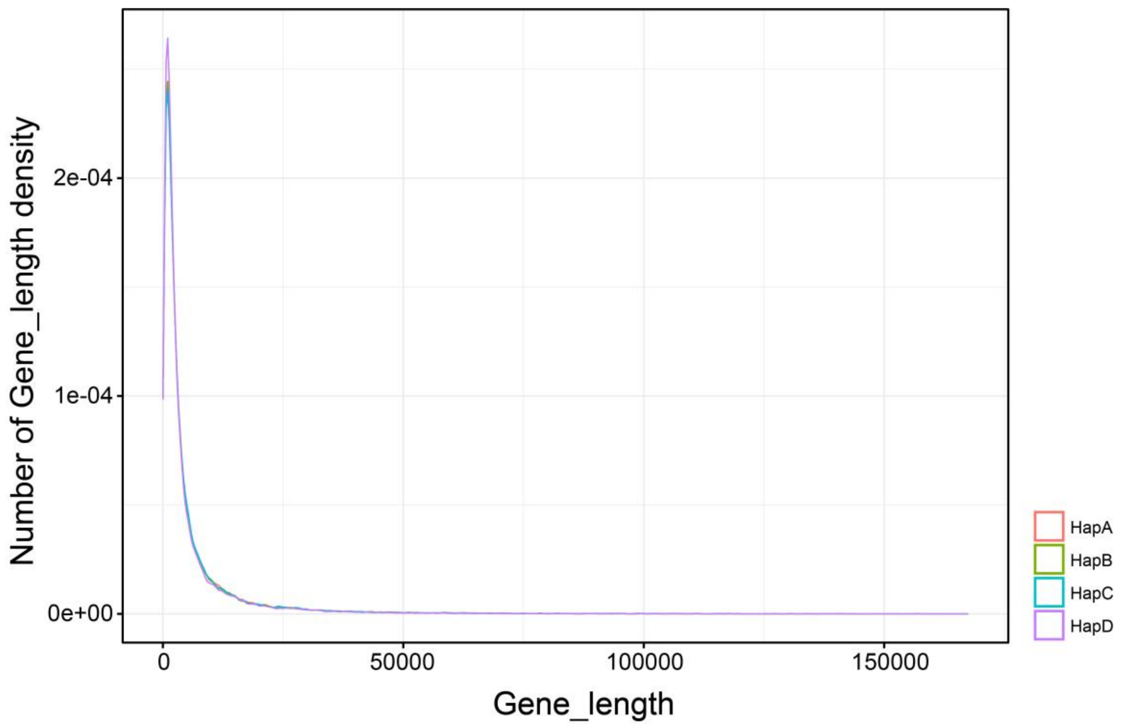

Supplemental Figure 11. Distributions and comparison of gene length features among four haplotypes (HapA, HapB, HapC, and HapD) assemblies of *D. deyangensis*.

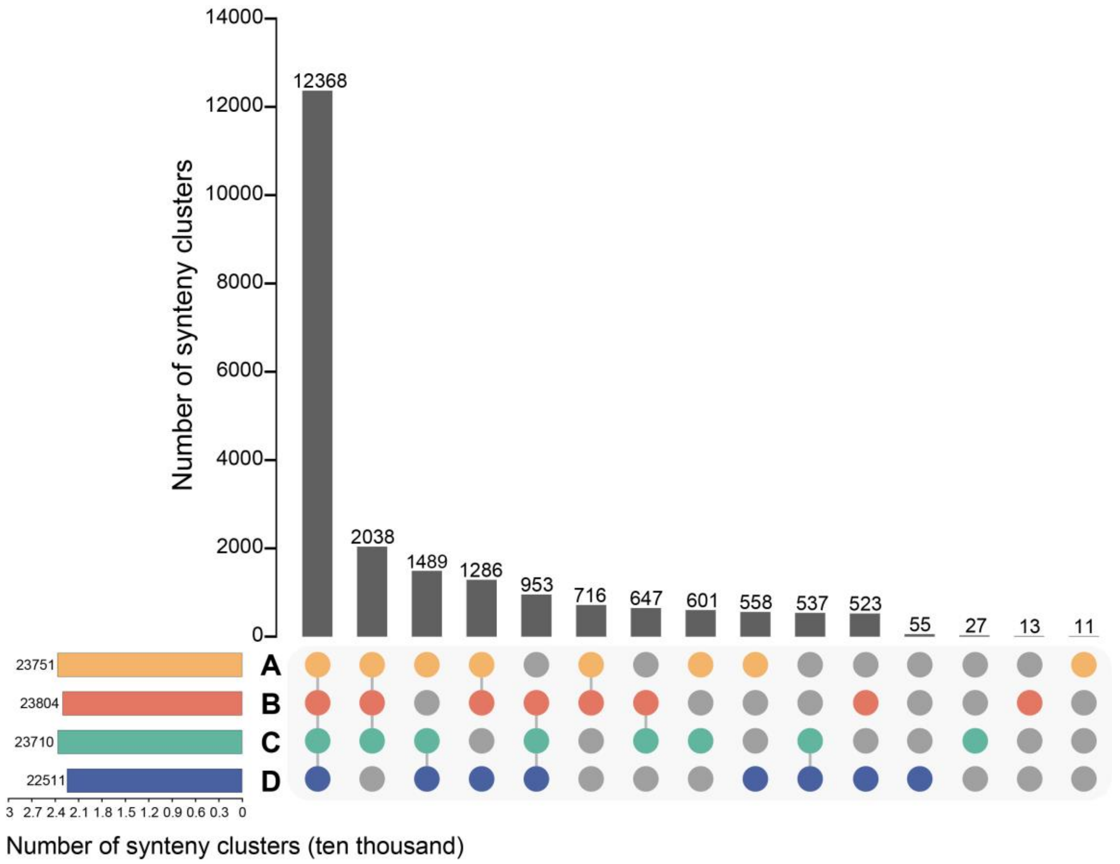

Supplemental Figure 12. Homology support of four monoploid genomes of *D. deyangensis* and shared gene synteny clusters among the monoploid genomes.

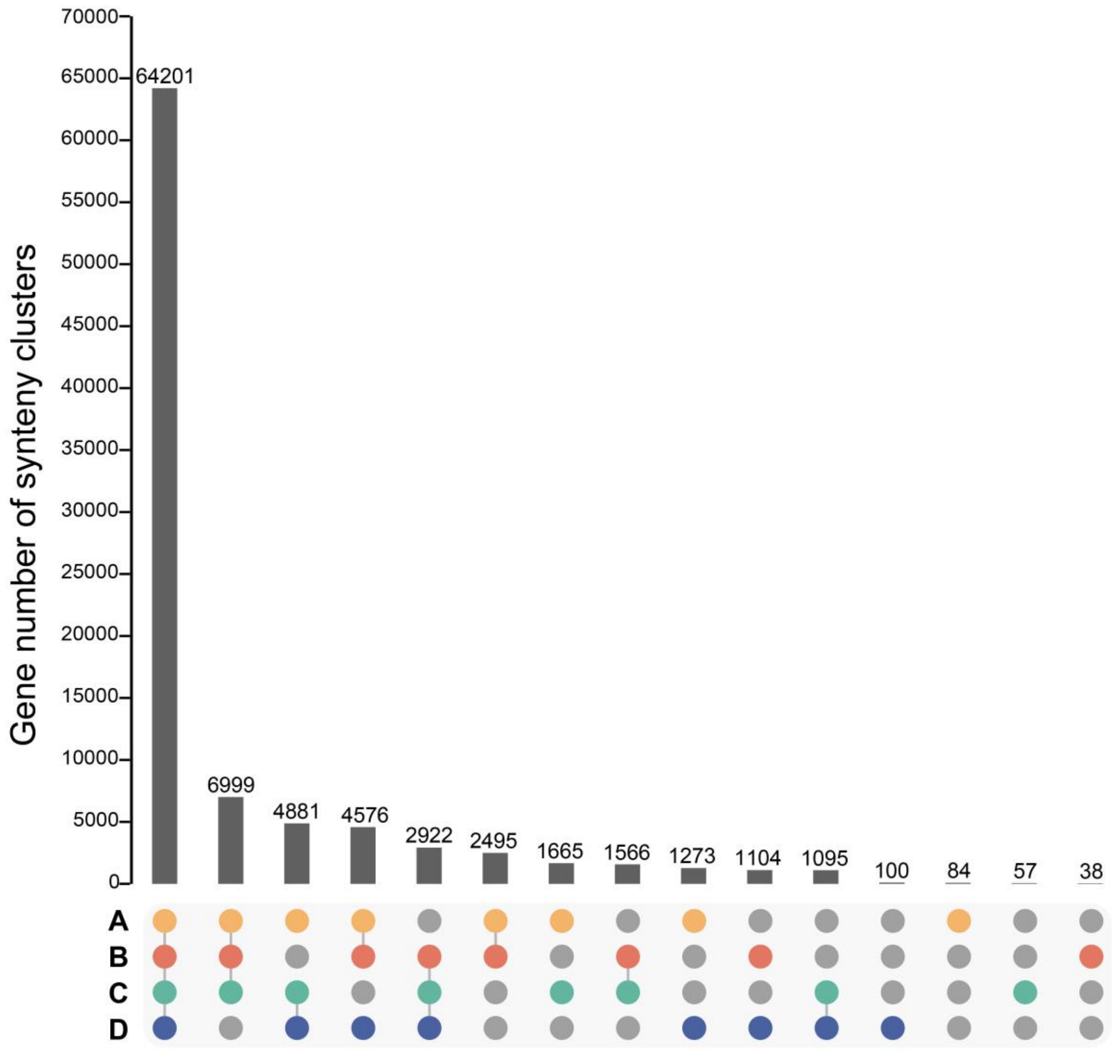

Supplemental Figure 13. Homology support of four monoploid genomes of *D. deyangensis* and shared gene number of synteny clusters among the monoploid genomes.

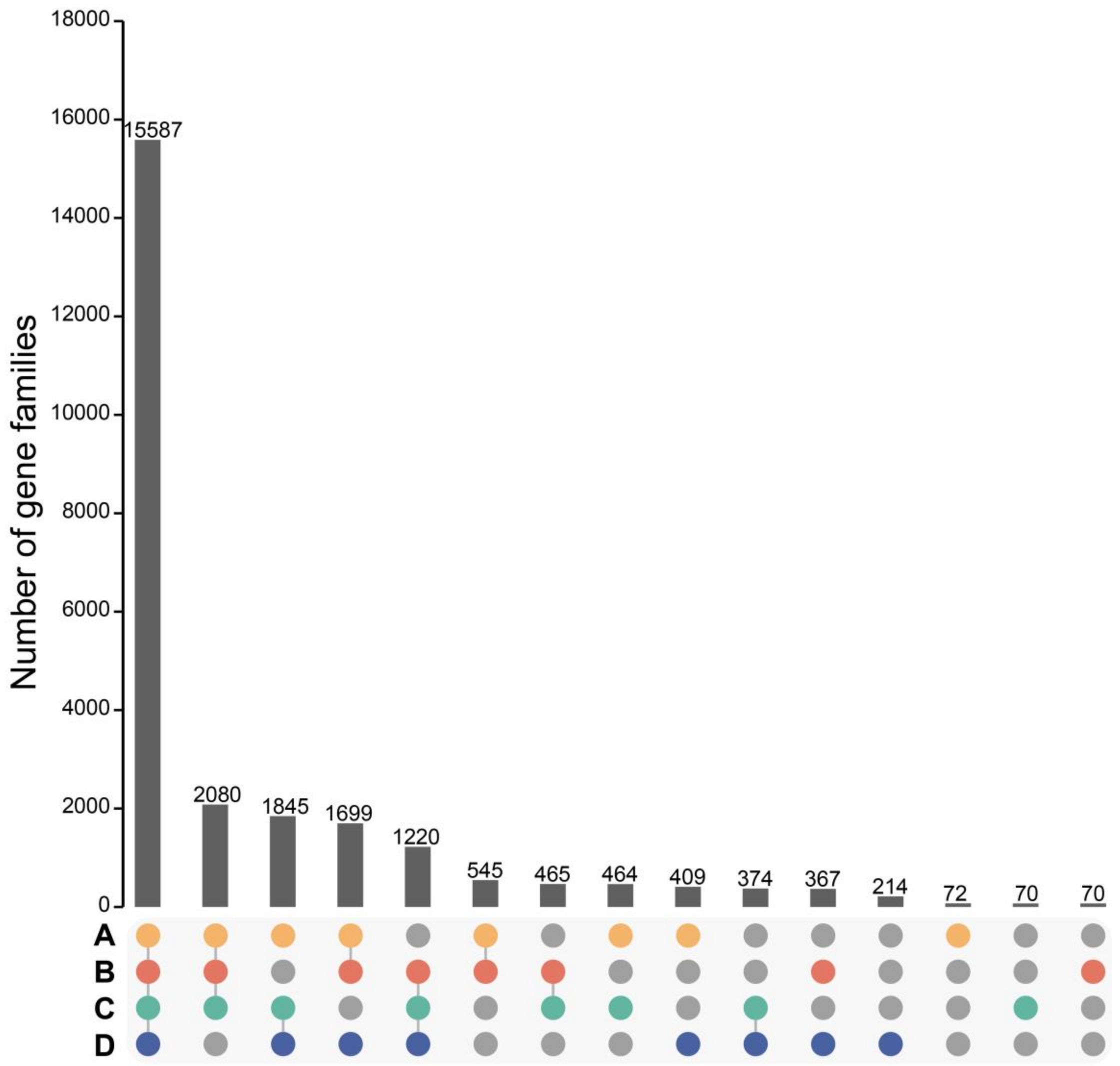

Supplemental Figure 14. Homology support of four monoploid genomes of *D. deyangensis* and shared gene families among the monoploid genomes.

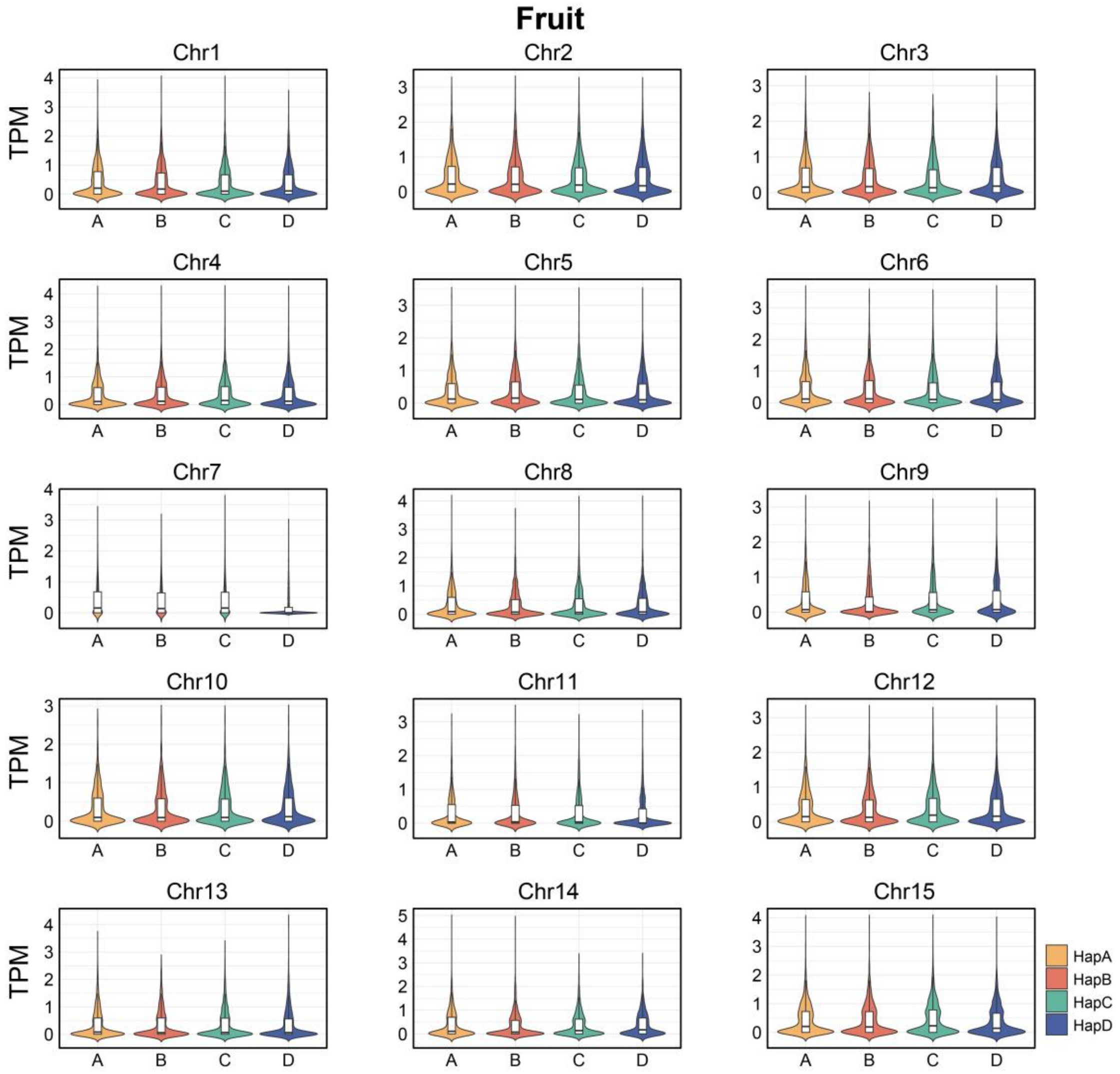

Supplementary Figure 15. Expression levels of genes in fruit tissue among four haplotypes (HapA, HapB, HapC, and HapD) in each chromosome groups of *D. deyangensis*. Transcripts per million (TPM values) gene expression values were computed using Kallisto v.0.46.2 and were log10-transformed for easier interpretation.

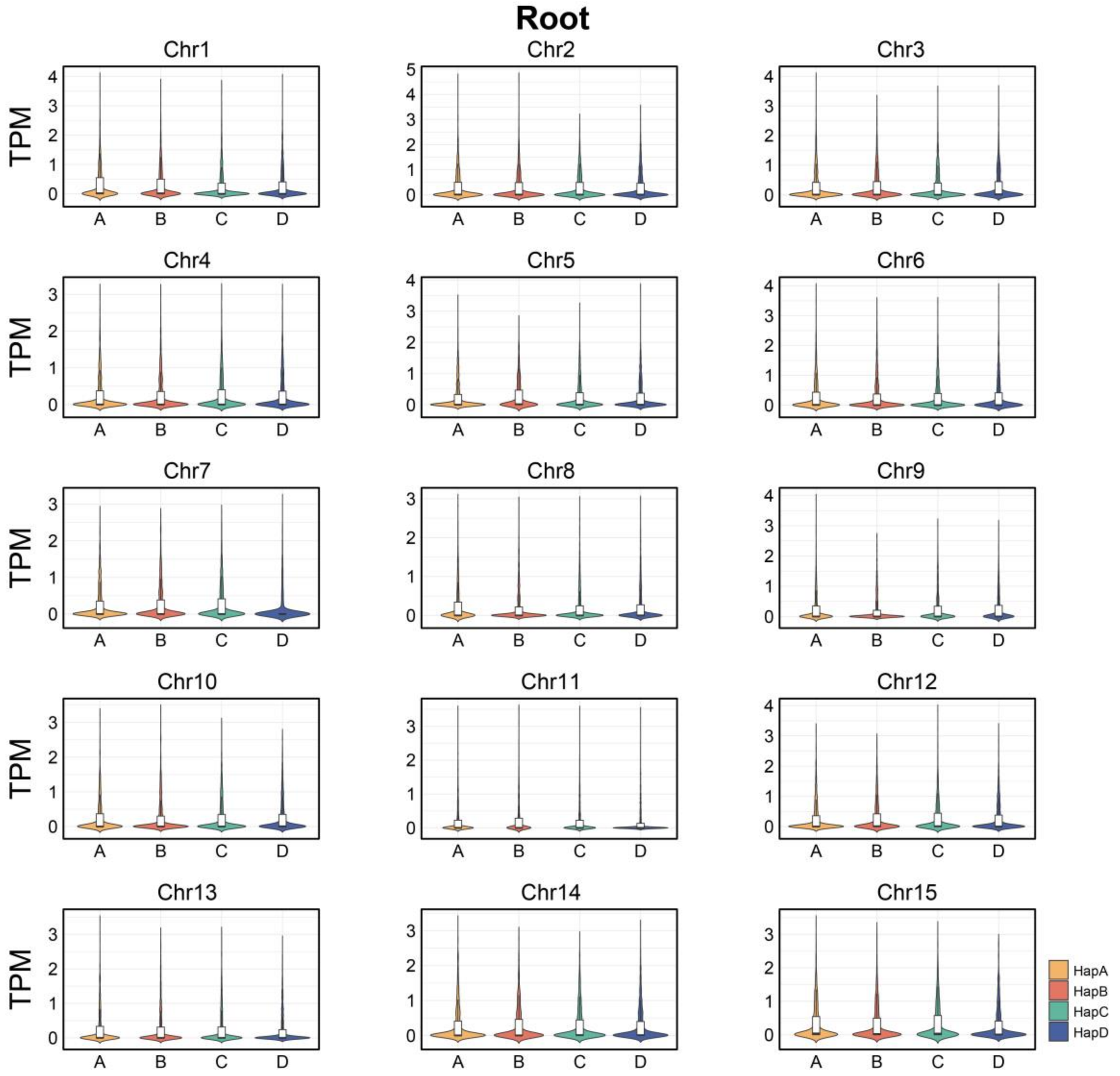

Supplementary Figure 16. Expression levels of genes in root tissue among four haplotypes (HapA, HapB, HapC, and HapD) in each chromosome groups of *D. deyangensis*. Transcripts per million (TPM values) gene expression values were computed using Kallisto v.0.46.2 and were log10-transformed for easier interpretation.

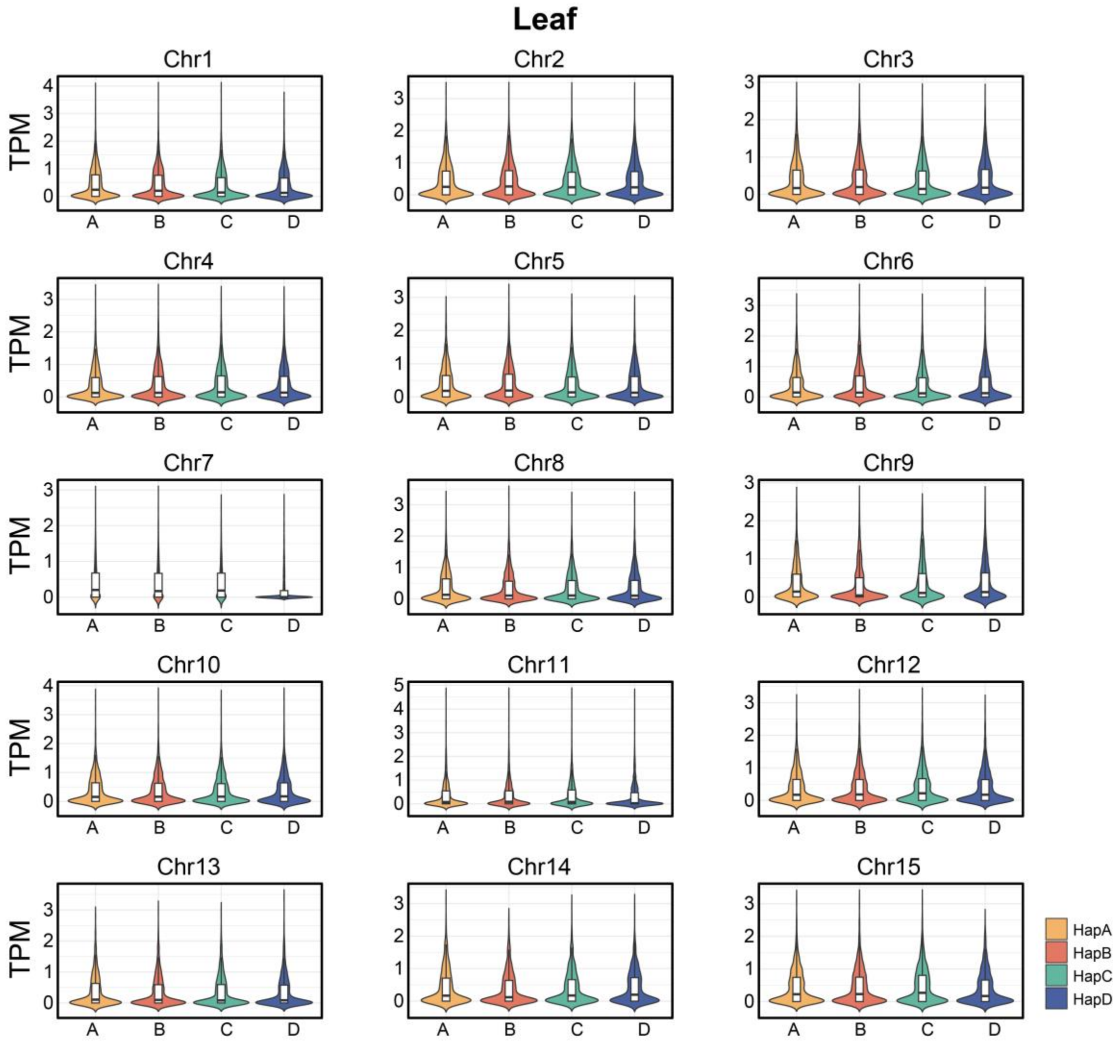

Supplementary Figure 17. Expression levels of genes in leaf tissue among four haplotypes (HapA, HapB, HapC, and HapD) in each chromosome groups of *D. deyangensis*. Transcripts per million (TPM values) gene expression values were computed using Kallisto v.0.46.2 and were log10-transformed for easier interpretation.

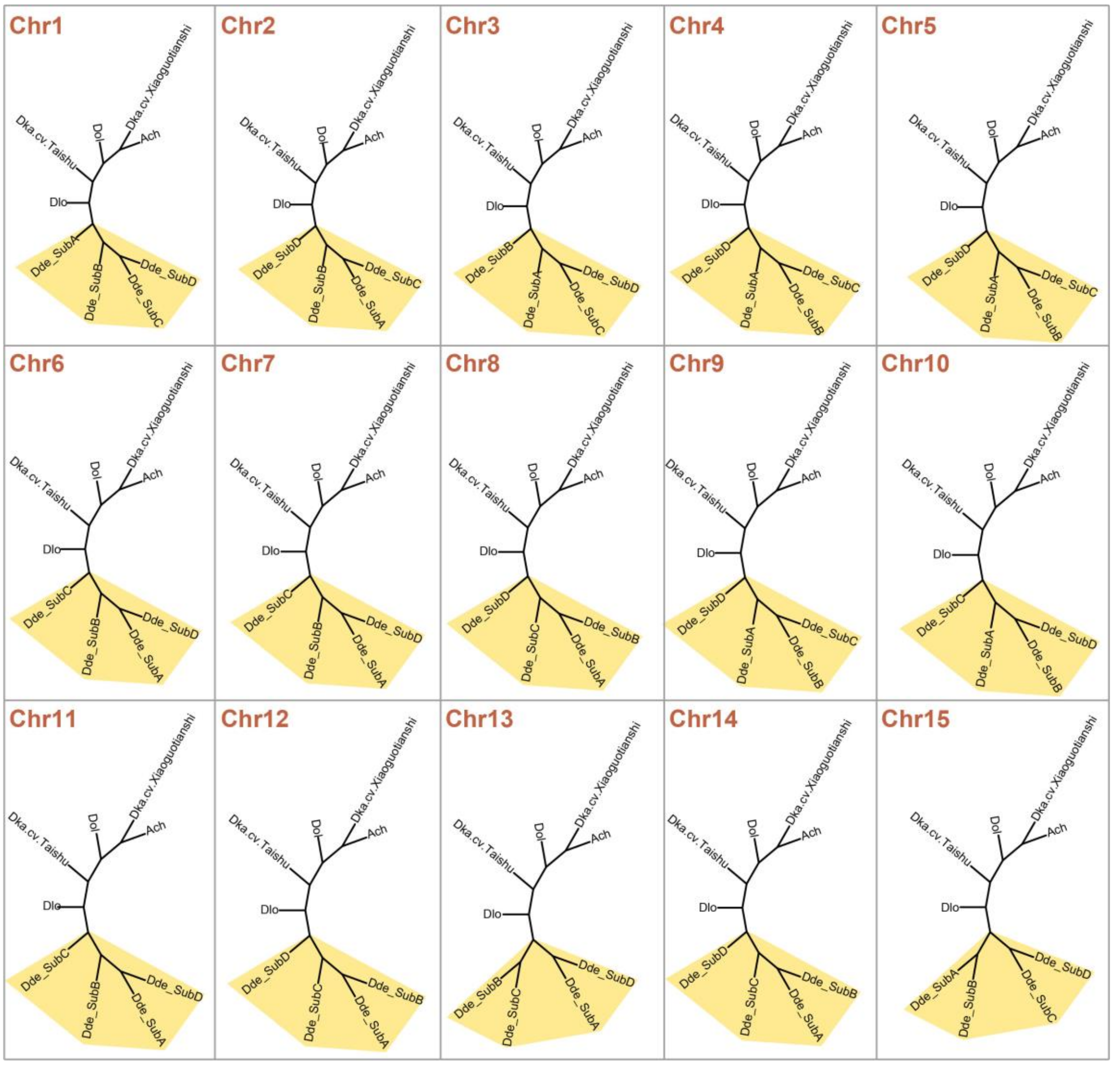

Supplemental Figure 18. Phylogenetic tree of 5 *Diospyros* sequenced species based on the fasta sequence of each chromosome, with *A. chinensis* (Ach) as outgroups. *D. deyangensis* (Dde_SubA/B/C/D), *D. lotus* (Dlo), *D. oleifera* (Dol), *D. kaki* (Dka), and *A. chinensis*.

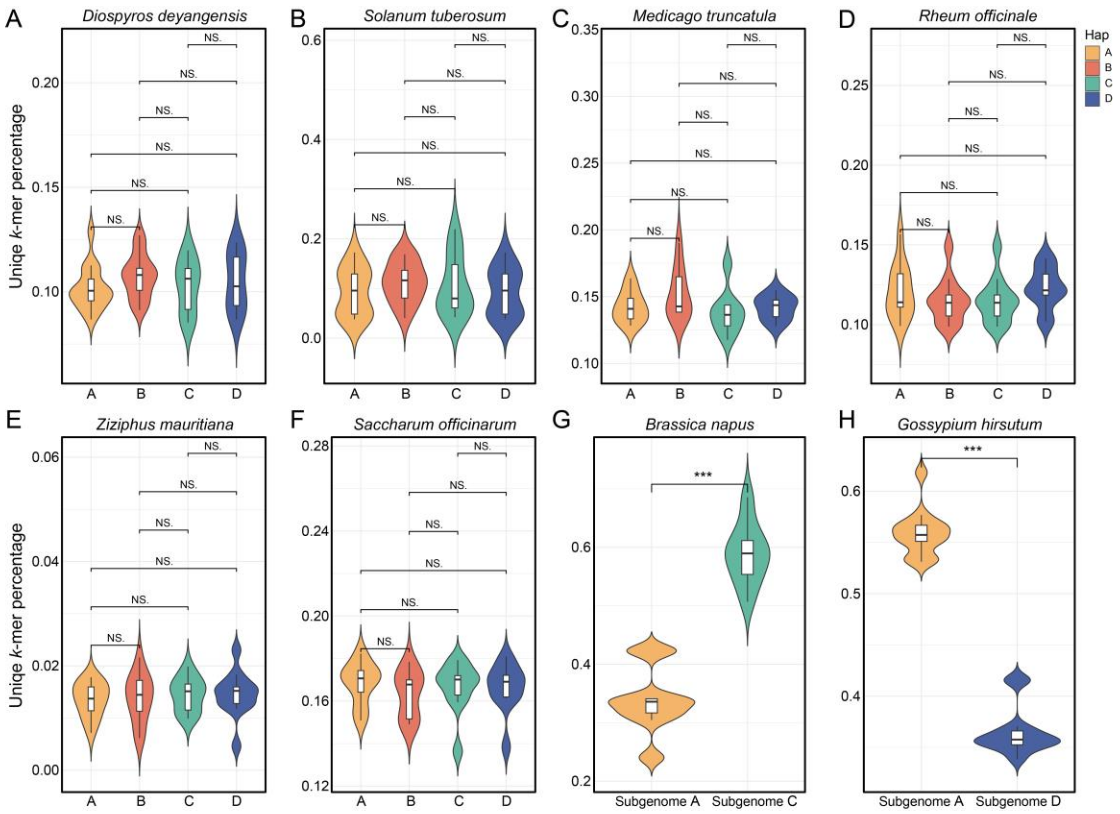

Supplemental Figure 19. Unique *k*-mer percentage of homologous chromosome for autotetraploid species *Diospyros deyangensis*, *Solanum tuberosum*, *Medicago truncatula*, *Rheum officinale*, *Ziziphus mauritiana*, *Saccharum officinarum* (A-F), allotetraploid species *Brassica napus*, and *Gossypium hirsutum* (G-H). The centerline in each box represents the median. The x-axis displays the haplotype genome (HapA-D), and pairwise significance was determined using the Wilcoxon test.

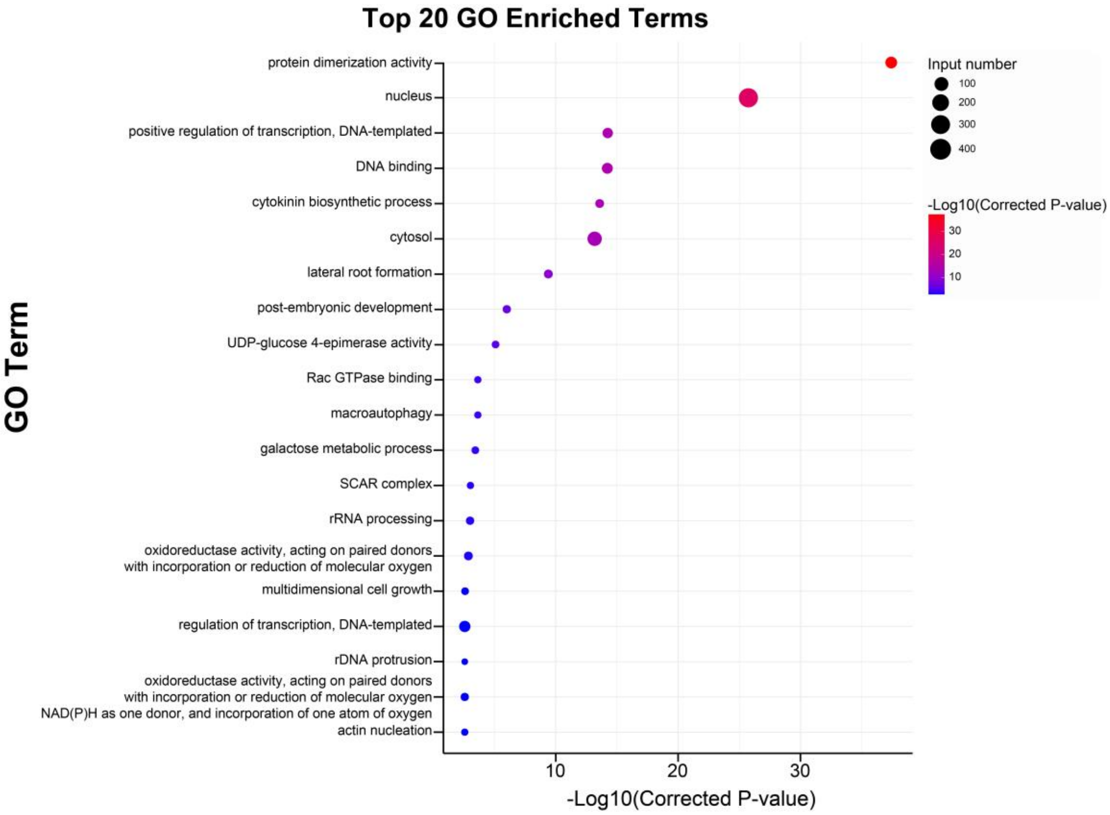

Supplemental Figure 20. GO enrichment analysis of the specific gene families in *D. deyangnsis*.

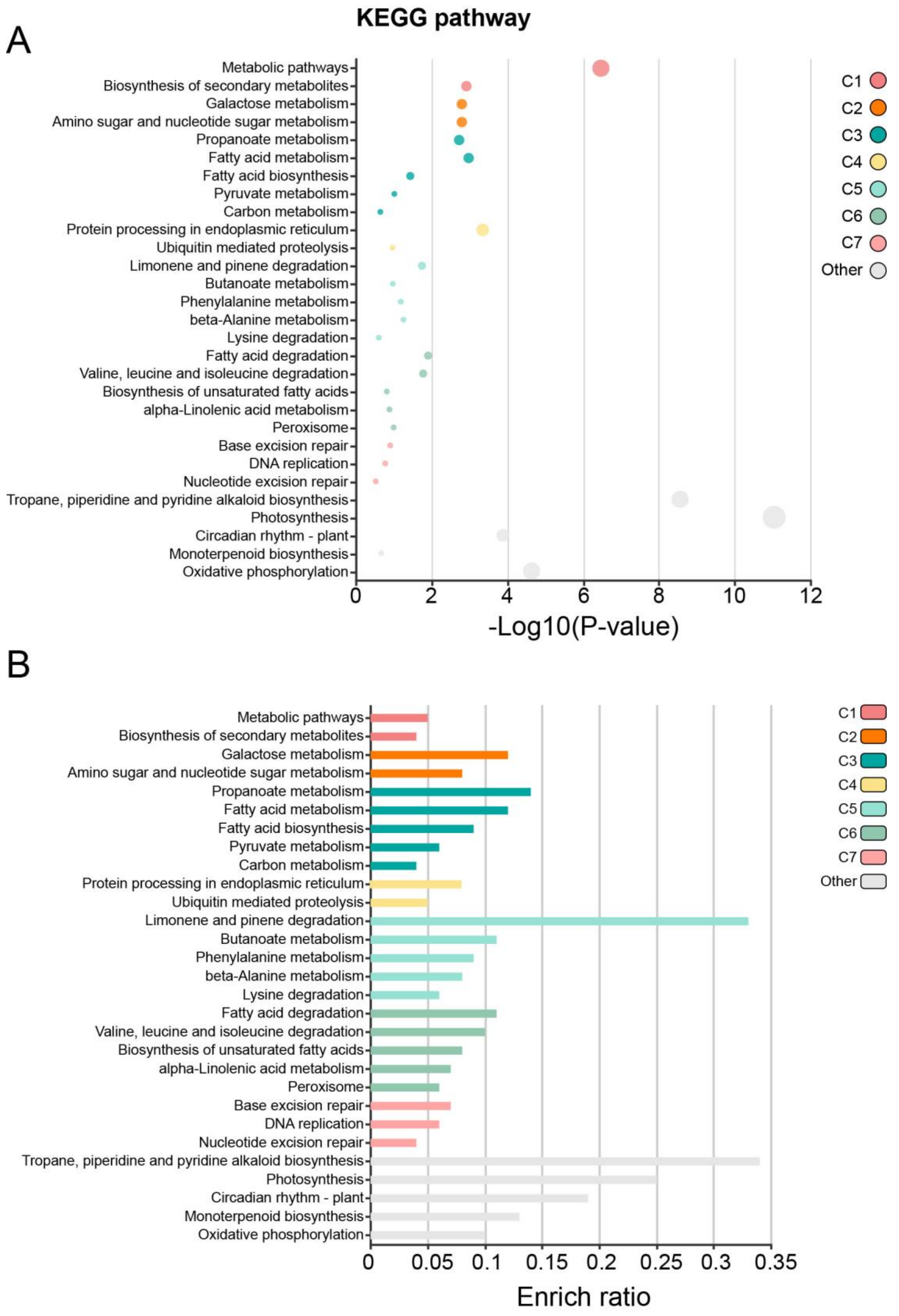

Supplemental Figure 21. KEGG pathway analysis of the specific gene families in *D. deyangnsis*.

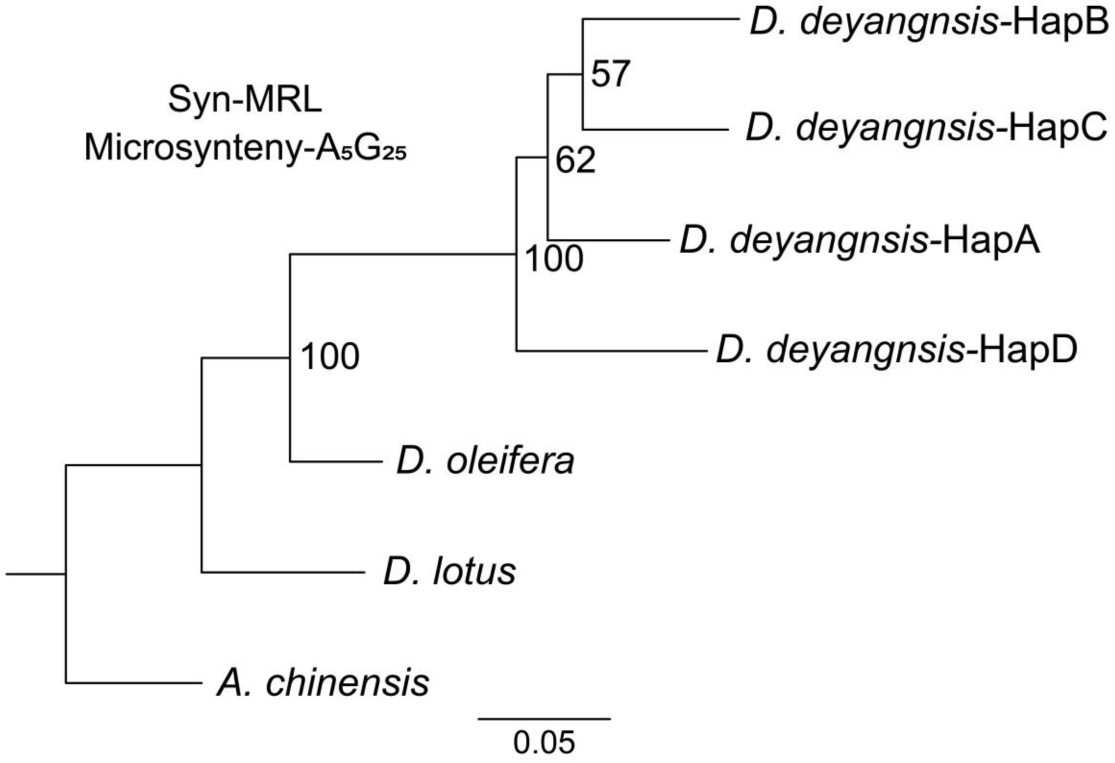

Supplemental Figure 22. Phylogenetic trees of *D.deyangnsis*, *D. oleifera*, *D. lotus* and *A. chinensis* (outgroup) obtained from whole-genome microsynteny (Syn-MRL). The panel shows phylogenies inferred by Syn-MRL under A_5G_{25} (A: the minimum number of anchor pairs required to call a collinear block in MCScanX, G: maximum number of intervening genes between two (adjacent) anchor pairs in collinear blocks in MCScanX).

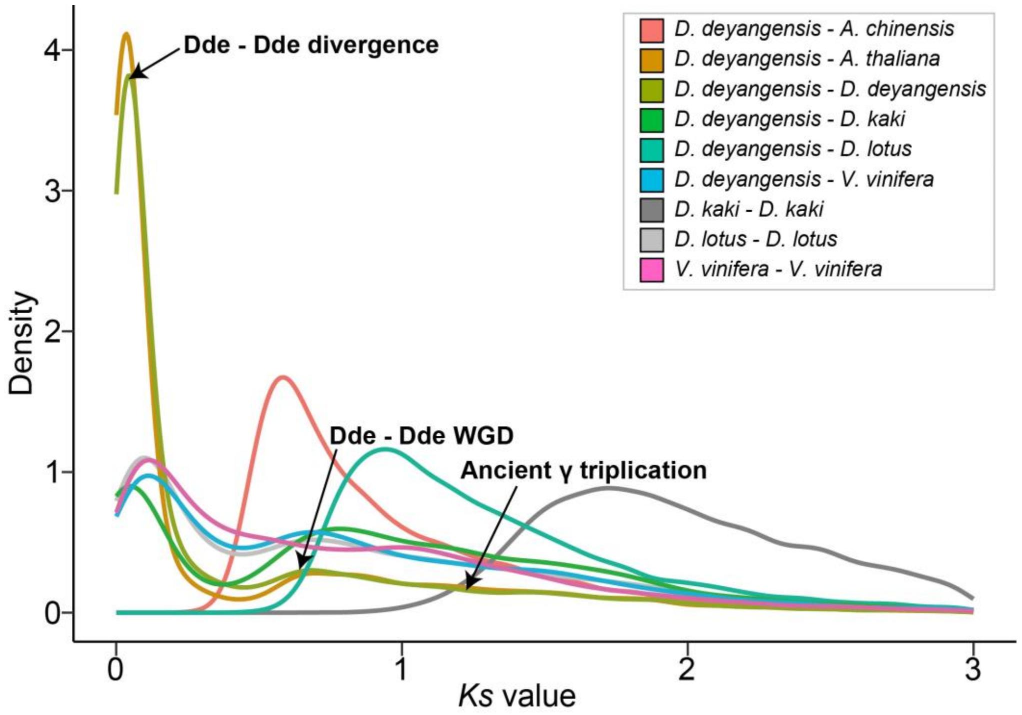

Supplemental Figure 23. *Ks* (Distributions of synonymous substitution rate) analysis of the 'Deyangshi' persimmon genome and other species of *Diospyros*.

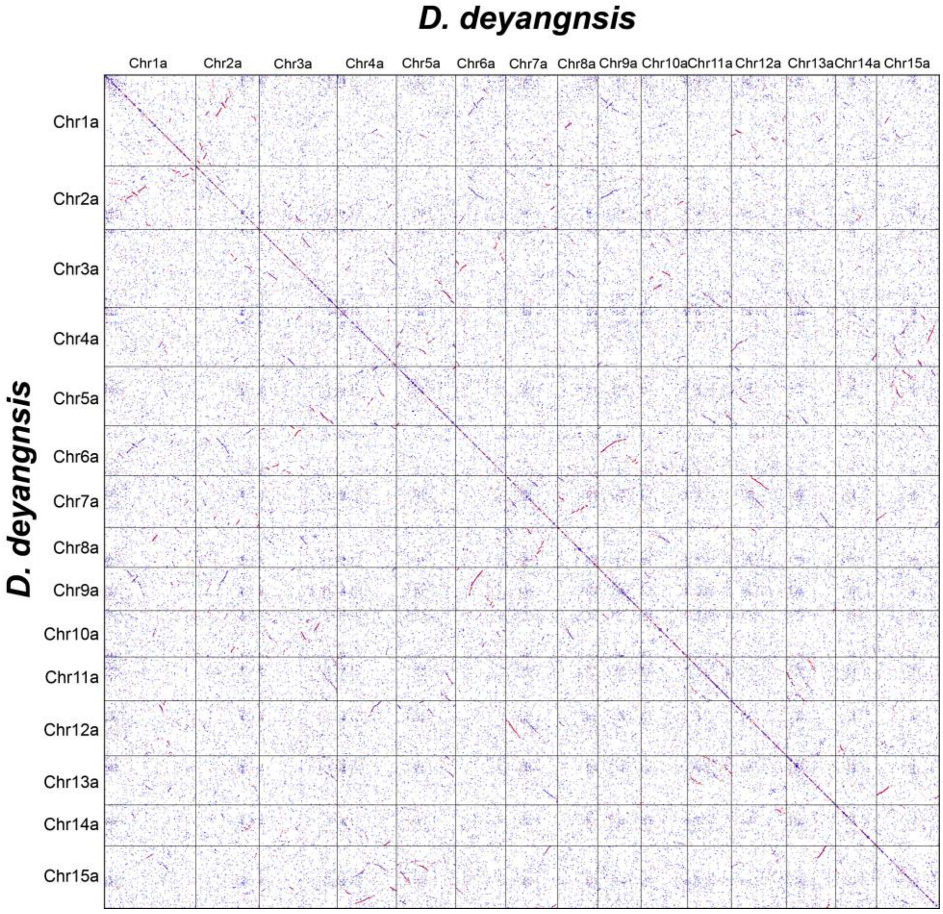

Supplemental Figure 24. Synteny dotplot of the *D. deyangensis* genome.

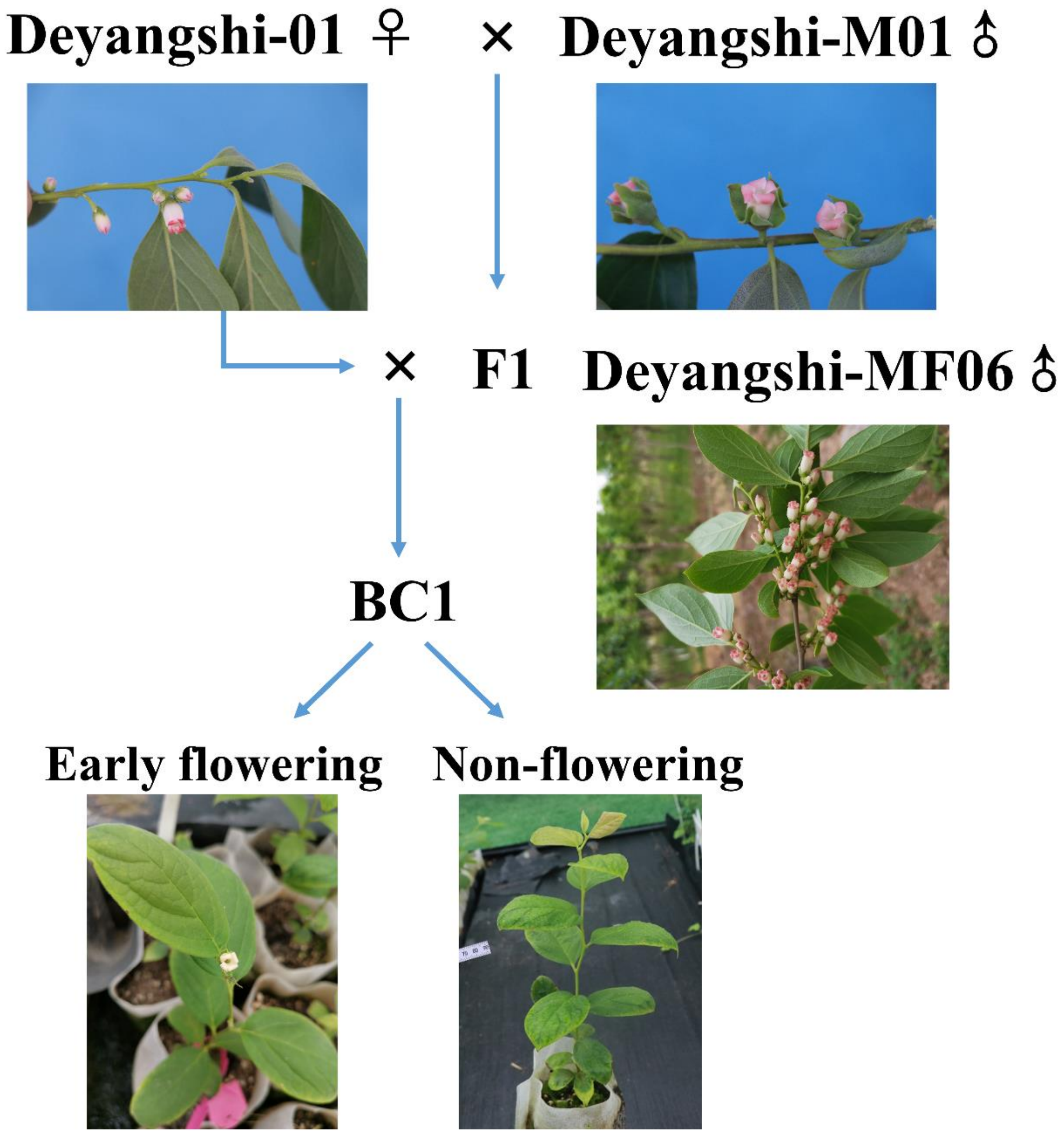

Supplemental Figure. S25. Construction of mapping BC1 population for BSR-seq in *D. deyangnsis*. Early flowering bulk (E-bulk) and non-flowering bulk (N-bulk) were composed of 50 individual plants respectively.
